# Magnitude processing and integration entail perceptual processes independent from the task

**DOI:** 10.1162/imag_a_00485

**Published:** 2025-02-24

**Authors:** Irene Togoli, Olivier Collignon, Domenica Bueti, Michele Fornaciai

**Affiliations:** Institut de recherche en sciences psychologiques (IPSY) et en Neurosciences (IoNS), Université catholique de Louvain, Louvain-la-Neuve, Belgium; Neuroscience Department, International School for Advanced Studies (SISSA), Trieste, Italy; HES-SO Valais-Walis, The Sense Innovation and Research Center, Lausanne and Sion, Switzerland

**Keywords:** magnitude perception, magnitude integration, EEG, numerosity perception, time perception, size perception

## Abstract

The magnitude dimensions of visual stimuli, such as their numerosity, duration, and size, are intrinsically linked, leading to mutual interactions across them. However, it remains debated whether such interactions, or “magnitude integration” effects, arise from perceptual processes that are independent from the task performed, or whether they arise from high-level decision-making processes. We address this question with two electroencephalography (EEG) experiments in which participants watched a series of dot-array stimuli modulated in numerosity, duration, and item size, in two separate conditions. In the “magnitude task” condition, participants judged either the numerosity, duration, or size of each stimulus. In the “contrast task” condition, instead, a separate group of participants performed a contrast oddball task, never attending or judging the magnitude of the stimuli. The results of the magnitude task first show robust integration effects across the three dimensions. Then, we compare the neural responses to magnitude across the two task conditions. This comparison shows very similar brain responses irrespective of the task, within a series of latency windows whereby the modulation of response amplitude can predict the behavioral magnitude integration effect (~150 and ~250 ms post-onset for numerosity and size; ~300 ms post-offset for the effect of duration). To better assess the similarity of brain responses to magnitude irrespective of the task, we use a cross-condition multivariate decoding analysis. This analysis demonstrates that brain responses in the magnitude task can predict the responses in the contrast task, at multiple latencies starting from early processing stages (~120 ms). These results suggest that magnitude processing and integration likely involve perceptual processes that are engaged irrespective of the task, thus independently from decision making, although the effect of duration on other magnitudes may also involve post-perceptual processes such as working memory.

## 1 Introduction

Magnitude dimensions such as numerosity, time, and space represent fundamental properties of the external world, as each of these dimensions provides essential information to understand and navigate the environment. Indeed, the perception of magnitudes organizes our thoughts and experience by allowing us to appreciate how many objects are around us, their size and their spatial relations, and the duration and timing of the external events. While these dimensions are important in their own rights and studied in separate lines of research, a particularly interesting phenomenon is their integration and interaction, possibly grounded on their shared computational structure (e.g.,Walsh, 2003). Different magnitude dimensions seem, indeed, linked in a way that the perception of one dimension depends on the others, usually leading to mutual biases. For instance, a large object or a numerous set of items is perceived as lasting longer in time compared to a smaller object or fewer items (e.g.,Xuan et al., 2007). Vice versa, a longer stimulus may appear bigger or more numerous than a shorter one (e.g.,Javadi & Aichelburg, 2012;Lambrechts et al., 2013;Togoli et al., 2021,^2024^).

These mutual influences across magnitude dimensions—or “magnitude integration” effects—represent one of the core phenomena characterizing magnitude perception, and have inspired important theories like the “a theory of magnitude” (ATOM) framework (Walsh, 2003). According to ATOM, the processing of different magnitudes culminates in a generalized magnitude system encoding different dimensions with the same neural code. This, in turn, would allow the interaction of magnitude information in the service of perception and behavior. This view has been, however, challenged by the idea that biases across magnitudes, and especially space and time, may stem from the linguistic labels assigned to them, and how we conceptualize these dimensions at the linguistic rather than perceptual level (“metaphoric theory”; e.g.,Casasanto & Boroditsky, 2008). While evidence has now been accumulated against a purely linguistic/conceptual view of magnitude integration (Cai & Connell, 2015;Togoli, Bueti, et al., 2022;Whitaker et al., 2022), other theories have proposed alternative cognitive mechanisms mediating magnitude interactions, based on working memory interference (Cai et al., 2018), or response biases (Yates et al., 2012). Namely, according to these ideas, the interference across magnitudes would occur either because of different memory traces nudging each other while stored in working memory, or because of a bias in the response selection due to the similar response codes of different magnitudes (i.e., “more” vs. “less”). In both these cases, the interference would not affect how magnitudes are perceived, but only their memory traces or the way they are judged. Finally, based on neuroimaging data, it has been recently proposed (Hendrikx et al., 2024;Tsouli et al., 2022) that the interaction could arise from the processing of different dimensions in partially overlapping cortical maps, especially in the parietal cortex (e.g.,Bueti & Walsh, 2009;Riemer et al., 2016,^2022^) but without involving a common neural code (Fortunato et al., 2023;Harvey et al., 2013,^2015^;Hendrikx et al., 2022,^2024^;Protopapa et al., 2019).

At which processing stage magnitude integration arises, thus, remains debated. Mixed evidence, indeed, seems to support both the “low-level,” perceptual interpretation, and the “high-level” interpretation based on memory and/or decision making. For example, results fromCai et al. (2018)show that duration judgments can be biased by the length of a stimulus only when the length information is provided before the duration judgment has started, suggesting that the bias occurs as an interference between memory traces. Furthermore, electroencephalographic (EEG) evidence fromCui et al. (2022)shows that the interference of length on duration is reflected by event-related potentials (ERPs) usually associated with working memory (i.e., the P2 and P3b component). Conversely, other results show that the integration effect does not occur every time two magnitudes are presented, as one would expect, for instance, from a response bias, but only when the two dimensions are conveyed by the same stimulus (e.g., a dot array with a given numerosity flashed to mark the onset and offset of a duration;Togoli, Bueti, et al., 2022). Instead, when two dimensions like duration and numerosity are conveyed by different stimuli (i.e., a texture marking the onset and offset of a duration, flashed on top of a dot array), the effect reverses becoming repulsive (i.e., the more numerous the stimulus is, the shorter it is perceived to last). This suggests that magnitude integration effects are not the result of a simple interference between different types of information, but involve perceptual binding processes.

To further assess the nature of the magnitude integration phenomenon, here we compare the neural (EEG) signature of magnitude integration when magnitudes are actively judged in a task, versus when they are passively watched while attending a different feature of the stimuli (i.e., contrast). In the first experiment, the participants judged either the numerosity, the duration, or the item size of dot-array stimuli against a reference presented before the start of each block (magnitude task condition). In a second experiment, a separate group of participants watched a similar stream of dot-array stimuli modulated in numerosity, duration, and item size, but were asked to attend and respond to the contrast of the stimuli (i.e., contrast oddball detection task; contrast task condition). Our hypothesis is that if magnitude processing and integration entail perceptual processes, then similar magnitude-sensitive neural signatures should be observed irrespective of whether the participants are actively judging magnitudes or not. Specifically, what we define as perception in this context includes the stream of processing that starts with early sensory encoding and culminates with a perceptual representation of a stimulus, before such a representation enters later post-perceptual stages involving for instance working-memory manipulation (e.g., comparison between a target stimulus and a memorized reference), decision, and response selection. According to this, a perceptual effect should involve a bias in the subjective appearance of a stimulus, and not just in its mnemonic trace when retrieved to perform a task, and should thus emerge irrespective of whether magnitude information is subject to working memory and decisional processes or not. Conversely, if the integration effect hinges upon active manipulation in memory or decision-making, then brain activity during the magnitude task should show a unique neural signature not generalizing to the contrast task condition, where the magnitudes are neither memorized nor judged. In other words, a mnemonic or decisional interference should show a signature specific to the condition where magnitudes are actively judged. To test this hypothesis, we first identify a neural signature of magnitude integration in the magnitude task condition, by assessing the extent to which the brain responses could predict the integration effect measured behaviorally. We then compare such a neural signature with brain activity evoked by the different magnitudes in the contrast task condition. Finally, to achieve a quantitative measure of how similar the brain responses in the two conditions are, we use a multivariate cross-condition “decoding” analysis. With this analysis, we thus assess the extent to which magnitude-sensitive brain responses during the contrast task can be predicted from the data of the magnitude task condition. If magnitude processing and integration entail similar mechanisms engaged irrespective of the task, then the brain responses to the magnitudes in the contrast task condition should be decodable based on the magnitude task data. Otherwise, if the task engages specific mechanisms resulting in different patterns of brain activity, no cross-condition decoding should be observed.

## 2 Materials and Methods

### 2.1 Participants

A total of 51 participants were tested in the study, with 20 participants tested in the magnitude task condition (13 females; age ± SD = 24.95 ± 4.21) and 31 separate participants tested in the contrast task condition (19 females; age ± SD = 23.96 ± 3.73). Two participants were excluded from data analysis in the contrast task condition due to corrupted EEG data files, leaving 29 participants included in the final analysis. Subjects were compensated for their participation in the study with 20 Euros. All participants read and signed a written informed consent form before the start of the session. All participants had normal or corrected-to-normal vision, and reported no history of neurological, psychiatric, or developmental disorders. The study was approved by the ethics committee of the International School for Advanced Studies (Protocol 10035-III/13), and was designed to be in line with the Declaration of Helsinki. The sample size tested in the magnitude task condition was determined a priori based on the expected magnitude integration effect as observed in previous studies from our group (Togoli, Bueti, et al., 2022;Togoli, Fornaciai, et al., 2022;Togoli et al., 2021). From these studies, we estimated an expected effect size (Cohen’s d) of 0.9. Note that this measure of effect size more liberally reflects an average estimate of the effect, rather than the minimum effect size observed in previous studies. We, however, also considered a more conservative level of power (90%), in line with previous studies from our group using similar experimental protocols (e.g.,Fornaciai et al., 2023). Thus, considering a power of 90% and a two-tailed distribution, the power analysis indicated a total estimated sample size of 16 participants, which we conservatively rounded up to 20. The sample size of the contrast task condition was instead chosen assuming a lower expected effect size (d = 0.55; seeFornaciai et al., 2023), reflecting the neural responses to magnitude as captured by a multivariate decoding analysis. Based on a one-tailed distribution and a power of 90%, a power analysis estimated a sample size of 30 participants. Note that the choice of using different distributions (two-tailed or one-tailed) in the power analyses of the two experimental conditions reflects the different underlying assumptions concerning the direction of the effect. Namely, in the magnitude task condition we considered possible behavioral effects in both the positive and negative direction (i.e., seeDeWind et al., 2015andFornaciai et al., 2019for negative effects between size and numerosity). In the contrast task condition, instead, the power analysis was based on the predicted results of a multivariate analysis of EEG data, for which we assumed results in only one direction (i.e., classification accuracy higher than chance level).

### 2.2 Apparatus and stimuli

The stimuli used in both conditions were arrays of black and white dots (50%/50% proportion), presented on a grey background at 90% of the maximum contrast. The white dots had a luminance of 88.4 cd/m^2^, the black dots 0.4 cd/m^2^, and the grey background had a luminance of 46.7 cd/m^2^. The stimuli were generated using the routines of the Psychophysics Toolbox (v.3;Kleiner et al., 2007;Pelli, 1997) in Matlab (r2021b, The Mathworks, Inc.), and presented on a 1,920 × 1,080 LCD monitor running at 120 Hz, which encompassed a visual angle of 48 × 30 deg from a viewing distance of 57 cm. The dot-array stimuli were generated online in each trial, with the dots scattered pseudo-randomly within a circular aperture with a variable radius spanning from 200 to 400 pixels (4.76 to 9.52 degrees of visual angle; pseudo-randomly determined in each trial). In the magnitude task condition, the dot-array stimuli could have a numerosity of 8, 12, 16, 24, or 32 dots, a duration of 100, 140, 200, 280, or 400 ms, and item size (i.e., the radius of each item in the array) of 3, 4, 6, 8, or 10 pixels (0.07, 0.09, 0.14, 0.19, and 0.24 degrees of visual angle, respectively), for a total of 125 unique combinations of the three magnitudes. The different ranges were designed to be approximately spaced in a Log2 scale. The reference stimulus that the participants used as a comparison (presented at the beginning of the session and before each block) had the middle value of the three ranges (16 dots, 200 ms, 6 pixels). The stimuli in the contrast task condition had smaller magnitude ranges to make the modulation of magnitudes subtler and less obvious, in order to better mask the true aim of the experiment. Namely, the stimuli could have a numerosity of 12, 16, or 24 dots, a duration of 140, 200, or 280 ms, and an item size of 4, 6, or 8 pixels (0.09, 0.14, 0.19 degrees of visual angle, respectively). Again, these ranges were designed to be approximately spaced in a Log2 scale. In both conditions, the stimuli were presented at the center of the screen.

### 2.3 Procedure

#### 2.3.1 Magnitude task condition

**Fig. 1.**
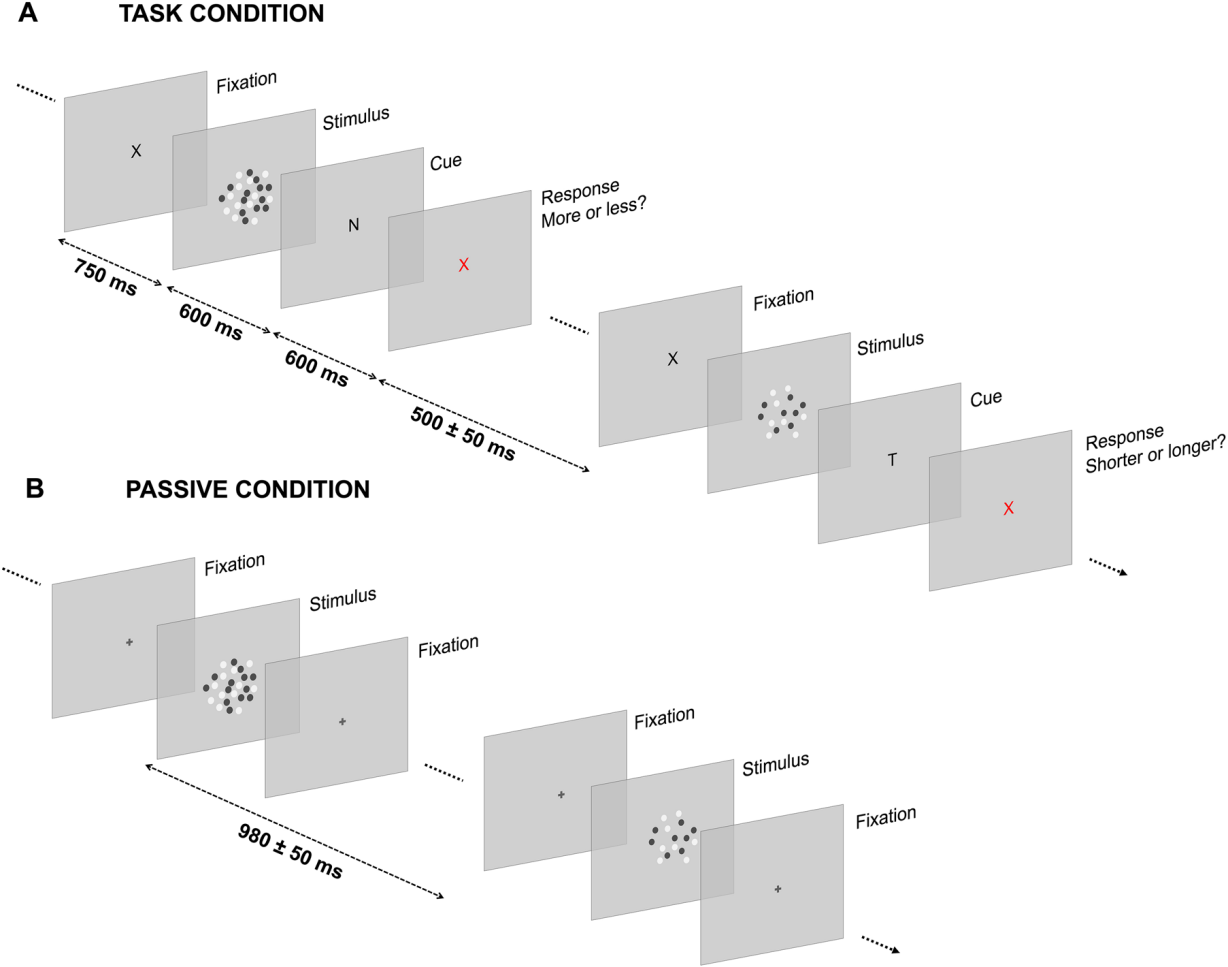
Experimental procedure. (A) Procedure of the magnitude task condition. While participants kept their gaze at the center of the screen (on a “X” that served as fixation cross), a stimulus was presented in each trial. The stimulus was modulated in numerosity (8–32 dots), duration (100–400 ms), and item size (i.e., the size of each item in the array; 3–10 pixels). After an interval of 600 ms from the offset of the stimulus, a cue appeared at the center of the screen indicating which stimulus dimension the participant had to judge. Namely, the cue could be an “N” (numerosity judgment), a “T” (i.e., “time”; duration judgment), or an “S” (size judgment). The cue remained on the screen for 600 ms, after which a red fixation cross appeared on the screen, indicating to provide a response. The participants were then asked to report whether the magnitude dimension indicated by the cue was bigger or smaller compared to a reference corresponding to the middle of the magnitude ranges (presented before the session and before each block of trials). After providing a response, the next trial started after a variable inter-trial interval (500 ± 50 ms). (B) Contrast task condition. In the contrast task condition, participants watched a stream of dot-array stimuli modulated in numerosity (12–24 dots), duration (140–280 ms), and item size (4–8 pixels), while keeping their gaze on a central fixation cross. To ensure that participants watched the stimuli, they were asked to detect an occasional oddball stimulus (i.e., a dot array with reduced contrast) presented on 3.7% of the trials. Each stimulus was separated by a variable inter-stimulus interval of 980 ± 50 ms. Stimuli are not depicted in scale.

In the magnitude task condition (Fig. 1A), participants performed a magnitude classification task, with the dimension judged in each trial determined by a retrospective cue (i.e., presented after the offset of the stimulus). First, participants watched a reference stimulus representing the middle of the magnitude ranges used in the experiment and were instructed to remember it and judge the stimuli in the main sequence based on it. The reference was presented 10 times (randomizing the positions of the dots) before the start of the session and repeated 5 more time before the start of each block of trials. In the session, participants were instructed to keep their gaze on a central fixation point. Each trial started with the presentation of the fixation cross (an “X” at the center of the screen). After 750 ms, the dot-array stimulus was presented replacing the fixation cross, and was displayed for 100–400 ms according to the duration selected in the trial. After an interval of 600 ms from the offset of the stimulus, the retrospective cue was presented at the center of the screen. The cue could be either “N,” “T,” or “S,” respectively instructing the participant to judge the numerosity, duration (i.e., time), or item size of the stimulus. The cue remained on the screen for 600 ms. After that, the cue was replaced by a red X (fixation cross), instructing the participant to provide a response. According to the cue, the participant was asked to indicate whether the stimulus had a higher or lower numerosity, a longer or shorter duration, or a bigger or smaller item size compared to the reference. The response was provided by pressing either the down arrow (lower/shorter/smaller) or the up arrow (higher/longer/bigger) on a standard computer keyboard. After providing a response, the next trial started after a variable inter-trial interval of 500 ± 50 ms. Each participant completed a total of 1,250 trials (10 blocks of 125 trials), corresponding to a total of 10 repetitions of each unique combination of numerosity, duration, and dot size (i.e., 5 numerosities × 5 durations × 5 sizes × 10 repetitions). The three tasks were randomly intermixed within the same blocks. No feedback was provided to participants about their response.

#### 2.3.2 Contrast task condition

In the contrast task condition (Fig. 1B), the participants watched a series of stimuli modulated in numerosity, duration, and size. Each stimulus was presented centrally on the screen, and successive stimuli were separated by an inter-stimulus interval of 980 ± 50 ms. In order to make participants attend the stream of stimuli, they were asked to detect occasional oddball stimuli defined by a reduced contrast compared to the rest of the stimuli (30% contrast instead of 90%; contrast oddball detection task). The oddball stimuli represented 3.7% of the total stimuli presented. Participants were thus instructed to press the space bar on the keyboard as fast as they could once they detected an oddball stimulus. This occasional simple detection task was designed to avoid drawing attention to any of the magnitude dimensions of the stimuli, while encouraging the participants to watch the stimuli. On average, the detection rates (±SD) of the oddball were 93% ± 1.3%, and the average reaction time was 313 ± 11 ms. In the contrast task condition, participants completed a total of 2,160 trials (8 blocks of 270 trials), for a total of 80 repetitions of each combination of stimulus magnitudes (i.e., 3 numerosities × 3 durations × 3 sizes × 80 repetitions). The higher number of trials tested in the contrast task condition compared to the magnitude task condition was chosen to compensate for the smaller magnitude ranges used, that is, in order to ensure that we could measure robust brain responses to the magnitudes also in this condition. Overall, participants were only instructed to watch the stream of stimuli and respond to the oddball, and the magnitudes were never mentioned in the instructions and recruiting materials.

Note that the stimuli and procedures of the two experimental conditions were partially different. When designing the experiments, indeed, we deemed it more important to have paradigms specifically tailored to each condition rather than using the exact same procedure, in order to increase the sensitivity of each experiment. Specifically, while a more extended magnitude range is necessary in the magnitude task condition in order to assess the behavioral effect of magnitude integration (seeSection 2.4below), such extended range is not necessary for the contrast task condition. In this latter condition, we thus preferred to have fewer levels of magnitude and increase the number of repetitions of each level to increase the signal-to-noise ratio of the EEG analysis, and smaller differences across different levels to provide a subtler modulation of the different dimensions. When it comes to the timing of stimulus presentation, the magnitude task condition requires the addition of blank intervals between the offset of the stimulus and the onset of the task cue and the response, to avoid introducing noise to the brain responses to the stimulus itself. Since those intervals are not needed in the contrast task condition, we chose a faster presentation rate to avoid having long blank periods, and to increase the number of trials that we could test during the experimental session. According to the hypothesis tested in this study (seeSection 1), such differences in the stimuli and the procedure should not affect the assessment of magnitude processing and integration and the comparison of brain responses to the magnitudes across the two experimental conditions.

### 2.4 Behavioral data analysis

In the magnitude task condition, the magnitude judgment performance and the integration effect were assessed by first computing the point of subjective equality (PSE). The PSE reflects the accuracy in the task, and the perceived magnitude of the stimuli compared to the reference magnitude. Data reflecting each specific task (i.e., all the trials in which a specific cue was presented) were used to compute the proportion of “more” (numerosity task), “longer” (duration task), or “bigger” (size task) responses as a function of both the task-relevant magnitude and the other (interfering) magnitudes. A psychometric (cumulative Gaussian) function was then fitted to the distribution of proportion of response, according to the maximum likelihood method (Watson, 1979). Specifically, within each task, the psychometric function was fitted separately for each level of each of the other “interfering” magnitudes, in order to assess the difference in PSE due to the task-irrelevant magnitudes. For example, when analyzing the performance in the numerosity task, the fit was performed separately for each level of duration and each level of item size. To account for errors unrelated to the magnitude of the stimuli and lapses of attention, a finger-error rate correction of 2.5% (Wichmann & Hill, 2001) was applied. This correction reduces the asymptotic levels of the fit by a proportion corresponding to the rate, in order to account for the random errors preventing the proportion of responses from converging to 0% and 100% at the lower and higher end of the range, respectively. The PSE was computed as the numerosity/duration/size level corresponding to chance level (50%) responses (i.e., the median of the psychometric curve). This procedure allowed us to compute individual measures of PSE for the different combinations of task-relevant and interfering dimensions (e.g., numerosity PSE when duration was 100, 140, 200, 280, and 400 ms, and the same for size, and similarly for the other tasks). When performing the fit according to a given task-relevant and interfering magnitude, the other dimension was collapsed. Doing so, each data point in the fitting procedure represented the average of 25 repetitions of the specific combination of task-relevant and interfering dimension. Additionally, we assessed the precision in the task in terms of the Weber fraction (WF). The WF was computed as the ratio between the just noticeable difference (JND; the slope of the psychometric curve) and the PSE. To assess the difference in the average WF across the three types of tasks, we used a one-way repeated-measures ANOVA.

In order to better define the effect of the interfering magnitudes in each task, we then used the PSE to compute a “magnitude integration effect” index according to the following formula:

$$\begin{array}{l} \text{Magnitude Integration Effect}\\ \;\;\;=-1\text{ x }\left(\left(\text{PSEj}-\text{PSEref}\right)/\text{PSEref}\right)\text{ x }100; \end{array}$$

Where*PSE_ref_*correspond to the PSE obtained when the interfering magnitude considered was the same as the reference, and*PSE_j_*to the PSE corresponding to each other level of the interfering magnitude (either lower or higher than the reference). The change in sign (-1) was added in order to make the interpretation of the index easier. Namely, doing so a positive index means that the task-relevant magnitude is overestimated, while a negative index means that the magnitude is underestimated. To assess the significance of magnitude integration effects, we performed a series of linear mixed-effect model tests, assessing the integration biases on each type of judgment. Namely, we entered the magnitude integration effect as dependent variable, the ratio of each level of the interfering magnitude with the reference value and the magnitude itself (i.e., “numerosity,” “duration,” or “size”) as predictors. The subject, as well as the ratio and magnitude type, were also added as random effects (Magnitude integration effect ~ Ratio + Magnitude + Ratio x Magnitude + (1|subj.) + (1|Ratio) + (1|Magnitude) + (Ratio x Magnitude|subj.)). Interactions found between ratio and magnitude were followed up with additional LME tests within each interfering magnitude dimension. The LME models were chosen in this case (i.e., instead of ANOVAs) as the ratio is a continuous variable.

Finally, in order to assess the relationship between ERPs and the behavioral effect, we computed a measure of ΔPSE. This measure reflected the difference in PSE between each level of the interfering dimension and the middle magnitude level corresponding to the reference. This was done to have a similar measure that can be related to the neural effect of different magnitudes (see below for more information about the ERP analysis). All the analyses and statistical tests on behavioral data were performed in Matlab (version r2021b).

### 2.5 Electrophysiological recording and pre-processing

In both the magnitude and contrast task condition, the EEG was recorded throughout the experimental session. EEG recording was performed by using the Biosemi ActiveTwo system (at 2,048 Hz sampling rate), and a 32-channel cap based on the 10–20 system layout. To better monitor artifacts due to eye blinks and movements, we recorded the electro-oculogram (EOG) by means of a channel attached below the left eye of the subject. Since the BioSemi system employs active electrodes, the output signal had an impedance < 1Ω. Due to the negligible impedance of the active electrode system, the signal quality before and during recording was assessed by using the “electrode offset” measure, that is, the difference in signal amplitude between each electrode and the control electrode (CMS). During the recording, we made sure to keep the electrode offset values as low as possible. Usually, electrode offset values were kept in the range of ±20 µV, in line with the manufacturer (BioSemi) recommendations, but occasionally values up to ±30 µV were tolerated due to the limited time available to complete the procedure.

The pre-processing of EEG data was performed offline in Matlab (version R2021b), using the functions of the EEGLAB (Delorme & Makeig, 2004) and ERPlab (Lopez-Calderon & Luck, 2014) toolboxes. In both conditions, the pre-processing involved the binning and epoching of data according to each unique combination of the different magnitudes. In the magnitude task condition, the binning was also performed separately for the three cues determining the specific task in each trial. Initially, the preprocessing of the magnitude task condition involved epochs spanning from -300 to 1,200 ms around the onset of the stimuli, while the epochs used for the contrast task condition spanned a smaller range, from -200 to 700 ms, due to the different timing of the stimuli. During data analysis, however, we restricted the epochs of the magnitude task condition to -200:700 ms, as in contrast task condition. In the case of duration, ERPs were later re-aligned to the offset of the stimuli. This was done as we expected an effect of duration only after the presentation of the stimuli had fully unfolded. The pre-stimulus interval (-300:0 ms in the magnitude task condition, -200:0 in the contrast task condition) was used for baseline correction, subtracting the average pre-stimulus activity from the activity at each time point included in the epoch.

In both conditions, after the epoching, the EEG data were band-pass filtered with cut-offs at 0.1 and 40 Hz. Moreover, to clean up the data as much as possible from artifacts such as eye movements and blinks, we performed an independent component analysis (ICA) aimed at removing identifiable artifacts and other potential sources of systematic noise. After the ICA, we additionally applied a step-like artifact rejection procedure (provided by the EEGLAB toolbox;Delorme & Makeig, 2004), in order to exclude trials showing large differences in amplitude likely related to artifactual activity. This artifact rejection procedure involved an amplitude threshold of 40 μV, a window of 400 ms, and a step size of 20 ms, based on previous works from our group (e.g.,Fornaciai & Park, 2020;Fornaciai et al., 2017). This was done to further remove any remaining large artifact from the EEG data after the ICA correction. On average, this led to the exclusion of 2.26% ± 1.73% of the trials in the magnitude task condition, and 0.6% ± 1.2% in the contrast task condition. Finally, we computed the event-related potentials (ERPs) by averaging EEG epochs within each bin, and further low-pass filtered the signal with a 30-Hz cut-off.

### 2.6 Event-related potentials analysis

In both conditions, the ERP analysis was performed by considering the average of the same set of three occipital target channels, selected a priori based on previous studies (Fornaciai et al., 2017,^2023^;Tonoyan et al., 2022). The chosen target channels were Oz, O1, and O2. These channels, indeed, showed consistent magnitude-sensitive activity (at least for numerosity and duration) across several previous studies (e.g.,Fornaciai & Park, 2018;Fornaciai et al., 2017,^2023^;Park et al., 2016;Tonoyan et al., 2022), making them an ideal primary target for capturing the perceptual processing of the different magnitudes tested here.

First, we plotted the ERPs corresponding to each magnitude and computed the linear contrast of ERPs. The linear contrast represents the difference in amplitude of brain responses across the different levels of a given magnitude (e.g., seePark et al., 2016), and it is computed by multiplying the amplitude at each magnitude level by a specific weight. The weights of the linear contrast computation for each of the magnitude levels were [-2 -1 0 1 2] in the magnitude task condition, and [-1 0 1] in the contrast task condition, based on the number of levels of each magnitude tested in the two conditions. Moreover, we computed an additional measure of contrast by computing the difference in ERP amplitude for each level of each magnitude and the two extreme levels of the other two interfering dimensions. For example, for each level of numerosity we computed the difference in amplitude between the extreme levels of duration and size, and so on for the other dimensions. We then averaged this measure across the different levels of each magnitude. To assess the significance of the magnitude contrasts, we performed a series of one-sample t-tests against zero. To control for multiple comparisons, we applied a false discovery rate correction with q = 0.05. Finally, we computed a measure of ΔERP as the difference in amplitude between the ERPs corresponding to the middle level of the range (corresponding to the reference in the magnitude task condition) and each other level of the magnitude ranges. In the magnitude task condition, the ΔERP was computed separately according to the task. Doing so, we thus computed a measure of the effect of numerosity in the size and duration task, and so on for the other magnitudes. This measure was computed to have an index of the effect of magnitudes on ERPs similar to the ΔPSE computed from the behavioral results, in order to more easily relate the ERP and behavioral measures of the effect in the analysis of the magnitude task condition. To assess the significance of the ΔERP, in the magnitude task we performed a series of LME tests, entering ΔERP as the dependent variable, the ratio of each magnitude level and the middle level as predictor, and the subjects as well as the ratio as random effects (ΔERP ~ Ratio + (1|subj) + (1|Ratio) + (Ratio|subj)). In the contrast task condition, we instead performed a series of paired t-tests (i.e., due to the smaller number of levels of each magnitude range). In both cases, the tests were performed on small 10-ms windows (step = 5 ms) in a sliding-window fashion, and the significance was corrected with FDR (q = 0.05). Additionally, we considered significant only clusters of consecutive significant time-points larger than 10 ms (i.e., three consecutive significant tests or more).

Only in the magnitude task condition, we further assessed the relationship between ΔERP (i.e., neural effect of magnitude) and ΔPSE (i.e., behavioral effect of magnitude) via a series of LME tests. In this case, we entered the ΔPSE as the dependent variable, and the ΔERP as the predictor. The subjects as well as the ΔERP itself were also added as random effects (ΔPSE ~ ΔERP + (1|subj) + (1|ΔERP) + (ΔERP|subj)). This analysis was performed again across a series of 10-ms windows with step = 5 ms. Finally, the analysis was restricted to the latency windows showing a significant modulation of ΔERP by the magnitudes (see above), and we considered statistically significant only clusters of at least three consecutive significant windows. This analysis was aimed at assessing the extent to which magnitude-sensitive brain responses can predict the effect measured behaviorally.

### 2.7 Multivariate analysis

**Fig. 2.**
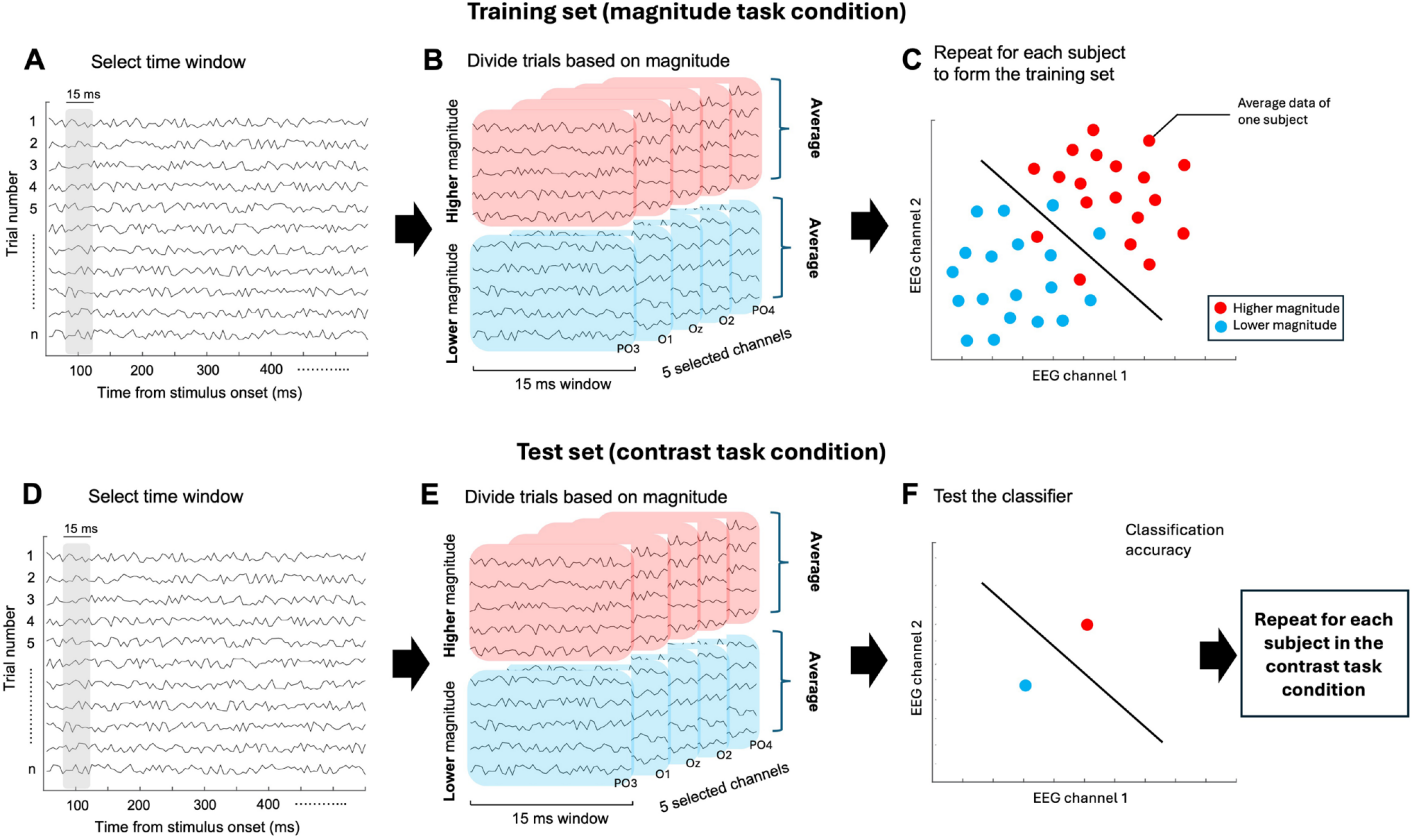
Multivariate analysis. Schematic depiction of the different main steps of the multivariate analysis across the two experimental conditions. (A) Selection of the time window. The analysis was performed iteratively across a series of 15-ms time windows (5-ms step) throughout the epoch. (B) The data of each individual participant in the magnitude task condition were sorted according to the magnitude presented, computing a measure of difference in response amplitude between higher or lower magnitudes and magnitudes corresponding to the middle value. The data within these two bins (“higher” and “lower” magnitude) were then averaged to obtain two datapoints for each participant, for each of the five features (EEG channels) used in the analysis. (C) The classifier was trained on a dataset including the average data of all the participants in the magnitude task condition, leaving out one datapoint for each class (i.e., training on 19 + 19 observations × 5 features). (D) The test dataset, based on the contrast task condition, was created by considering the same time window as the training set. (E) Considering the data of a single participant, we then binned higher and lower magnitudes and computed the difference in response amplitude compared to the middle magnitude level. Finally, we averaged the data within these two bins. (F) The classifier was tested on the average data of a single participant, with 1 + 1 observations × 5 features. This procedure was then repeated to consider each possible subset of training data, iteratively leaving out each data point. Finally, the entire procedure was repeated using data from each of the participants in the contrast task condition as test dataset, in order to obtain a set of classification accuracies equal to the sample size of the contrast task condition (N = 29). Note that panels C and F show an example of training and testing in a 2-dimensional space (i.e., two features, or EEG channels), while the actual procedure involved a 5-dimensional space based on the channels included in the analysis, which cannot be represented in the figure.

We relied on a multivariate approach in order to more directly compare the brain activity related to magnitude processing across the two conditions. Namely, we assessed the extent to which training a classifier on the data (ΔERP) from the magnitude task condition allows to decode magnitude-sensitive activity in the contrast task condition. A schematic depiction of the different steps of the analysis is shown inFigure 2. The procedure was designed to simulate a leave-two-out, cross-validated decoding procedure, but with the training and test set composed of data from different experimental conditions (and hence different groups of participants). The analysis was performed throughout the epoch, across a series of small time windows (15 ms, with a step of 5 ms) in order to increase the signal-to-noise ratio. Moreover, the features (i.e., EEG channels) included in the analysis were selected a priori, considering a larger set of channels compared to the ERP analysis. Namely, we chose the three channels previously used in the ERP analysis (O1, Oz, O2), with the addition of two nearby channels (PO3, PO4), for a total of five occipital and occipito-parietal channels. This choice is based on previous studies showing magnitude sensitive activity from both occipital and occipito-parietal channels (e.g.,Fornaciai et al., 2017;Park et al., 2016;Tonoyan et al., 2022). Additionally, the choice of including five channels was based on our experience with multivariate analyses in previous studies from our group, in which this number of channels represented a good trade-off between the amount of features entered into the analysis and the ability of the classifier to generalize the pattern of brain activity across independent datasets (i.e., avoiding over-fitting; e.g.,Fornaciai et al., 2023). The procedure was performed independently within each time window (Fig. 2A) and for each magnitude (numerosity, duration, size).

Within each time window (Fig. 2A), the first step was to create the training dataset, based on the data of the magnitude task condition. First, considering the data of a single participant, we sorted the trials according to the magnitude presented, and computed the difference between the amplitude relative to lower (8, 12 dots; 100, 140 ms; 3, 4 pixel) and higher (24, 32 dots; 280, 400 ms; 8, 10 pixel) magnitude levels and the amplitude of the middle magnitude level (16 dots, 200 ms, 6 pixel). Doing so, we obtained two bins of EEG data corresponding to a “higher” and a “lower” magnitude (Fig. 2B). We then averaged the data within the two bins to obtain two average data points for each of the five features included in the analysis. We repeated this step for each participant, in order to form the training dataset (for a total 20 data points for each feature and for each class entered into the analysis, equal to the number of participants in the magnitude task condition). According to the leave-two-out procedure, the classifier (support vector machine; C = 1) was then trained on a set of 19 + 19 datapoints (“higher” vs. “lower” magnitude, respectively; seeFig. 2C). The second step was to create the test dataset, based on the data of the contrast task condition. Considering the same time window (Fig. 2D), we again sorted the trials according to the magnitude and computed the difference in response amplitude between magnitudes either lower (12 dots, 140 ms, 4 pixel) or higher (24 dots, 280 ms, 8 pixel) than the middle level (16 dots, 200 ms, 6 pixel). Then, we computed the average within these two bins (Fig. 2E), for each of the five features, to create the test dataset. The classifier was then tested on these two datapoints corresponding to the average data of a single participant relative to the two classes of the analysis (“higher” and “lower” magnitude). This resulted in a measure of classification accuracy based on the ability of the classifier to classify the corresponding magnitude of each of the two datapoints. These training and testing steps were repeated in order to test all the possible subsets of the training datapoints (iteratively leaving out each datapoint in the training set), averaging the resulting classification accuracies. Then, this entire procedure was repeated for each participant in the contrast task condition, in order to obtain a set of classification accuracies equal to the number of participants in this condition, and for each time window throughout the epoch. Overall, each iteration of the analysis, within each time window and magnitude, was performed on a training dataset including 190 values (38 observations × 5 features) and a test dataset including 10 values (2 observations × 5 features).

To test for the significance of the distribution of classification accuracies obtained at each time window, we used a permutation (sign flipping) procedure. Namely, at each time window, we subtracted 0.5 from the classification accuracies (i.e., the chance level), and swapped the sign of half the values. This procedure was repeated 10,000 times taking random splits of the data, and we assessed the number of times that the classification accuracy of the sign-swapped data was equal or higher than the actual average classification accuracy observed. The proportion of times in which the simulated accuracy equaled or exceeded the actual value was considered the p-value of the test. The alpha level applied to these tests was 0.05. After the tests, we also applied a threshold of at least three consecutive time windows (i.e., only clusters of at least three consecutive time windows were considered significant). Note that we chose to use the contrast task condition data as the testing dataset in order to have a larger distribution of classification accuracy values to test with permutations, with the rationale of achieving more robust and stable results. We did not perform the analysis in the opposite direction (training with the contrast task data and testing with the magnitude task data) since the two sets of results would be difficult to average. Namely, due to the nature of this procedure, analyses in different directions would result in different distributions of classification accuracy values (29 when testing with the contrast task data, 20 when testing with the magnitude task data), making it difficult to combine them. All the analyses and statistical tests involving EEG data were performed in Matlab (version r2021b).

## 3 Results

In this study, we measured the neural (EEG) signature of magnitude processing and integration with subjects either engaged in actively judging the magnitude of the stimuli (magnitude task condition), or passively watching the modulation of different magnitudes while attending the contrast of the stimuli (contrast task condition). Doing so, we aimed at comparing such signatures to better understand the nature of the magnitude integration phenomenon. Specifically, if magnitude integration depends on post-perceptual cognitive processes, we predicted to observe a unique signature of magnitude processing only when performing a magnitude task. Conversely, if the integration effect arises from automatic perceptual processes, then similar signatures of magnitude processing should be observed in both the magnitude task and the contrast task.

### 3.1 Magnitude task condition

In the magnitude task condition, participants performed a magnitude classification task of the numerosity, duration, and item size of dot-array stimuli. Which dimension to judge was indicated to the participants via a retrospective cue (in a trial-by-trial fashion), thus forcing them to attend the stimulus as a whole rather than focusing on a single dimension. The procedure of the task condition is depicted inFigure 1A.

First, we assessed the behavioral effects of magnitude integration, which are shown inFigure 3A–C. To assess the mutual biases across the different dimensions, we first computed the point of subjective equality (PSE; seeSection 2.4) as a measure of accuracy in the task. Then, we computed a magnitude integration effect index based on the difference in PSE caused by each level of the interfering magnitudes compared to the reference magnitudes (i.e., the middle levels of numerosity, duration, and size). To assess the significance of magnitude integration, we performed a series of linear mixed-effect (LME) regression models within each type of task. In the model, we entered the magnitude integration effects as the dependent variable, the ratio of each interfering magnitude level with the reference level, and the magnitude itself (e.g., “duration” and “size” in the numerosity task), as predictors, and the subjects as well as the ratio and magnitude type as the random effects. The ratio (instead of the magnitude value) was chosen as a predictor to test the effect of both interfering dimensions on each type of judgment within the same test.

**Fig. 3.**
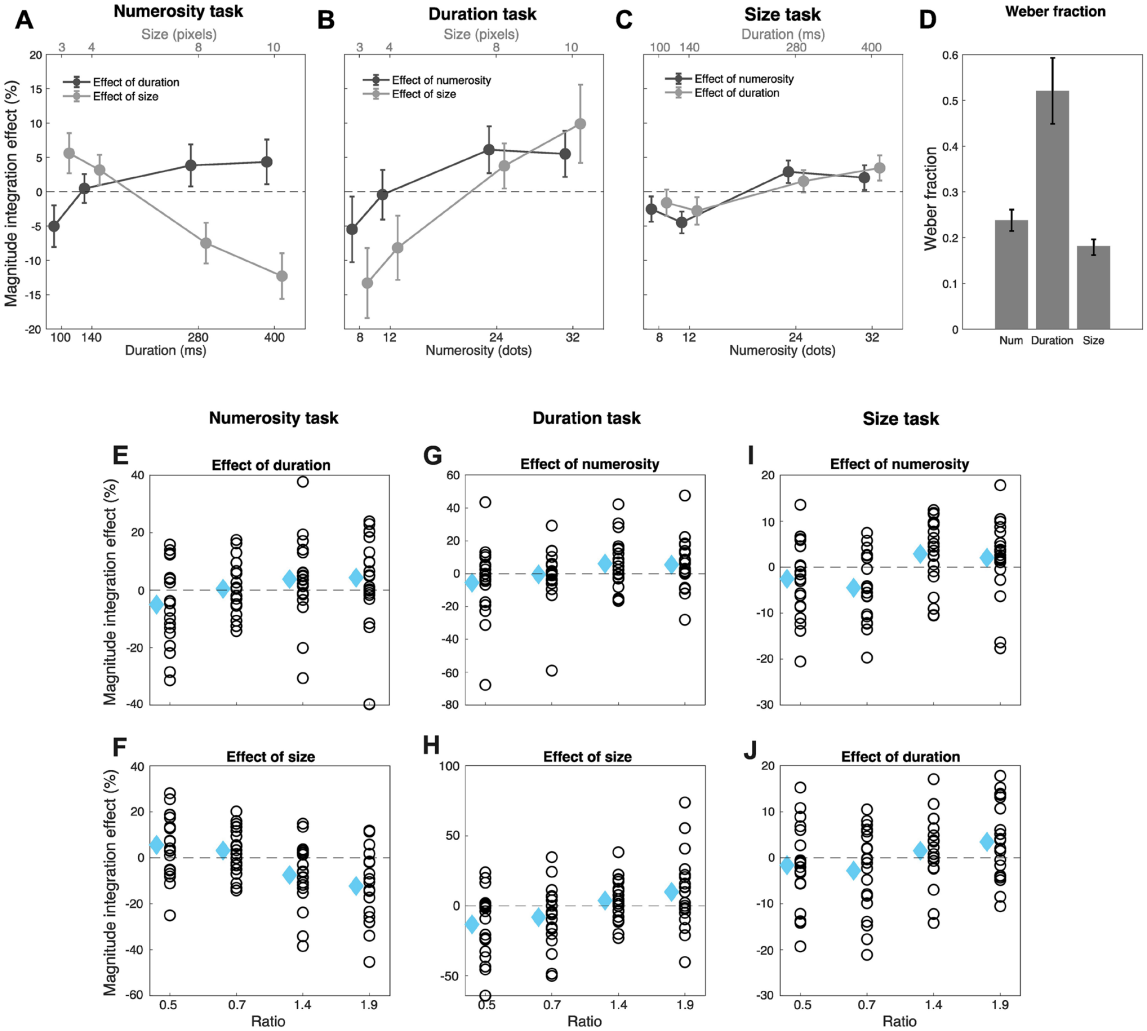
Behavioral effects of magnitude integration. (A) Magnitude integration effects of duration and size on numerosity judgments. The values of the two interfering dimensions are reported on the upper and lower x axes. (B) Magnitude integration effects on duration judgments. (C) Magnitude integration effect on size judgments. The data points are slightly jittered for the ease of visualization. (D) Average Weber fraction in the three tasks. Error bars are SEM. (E–J) Plots showing the individual measures of the magnitude integration effect across the group, as a function of the ratio between each level of magnitude and the middle level. (E) Plot of individual effects relative to the effect of duration in the numerosity task. (F) Effect of size in the numerosity task. (G) Effect of numerosity in the duration task. (H) Effect of size in the duration task. (I) Effect of numerosity in the size task. (J) Effect of duration in the size task. Note that the scale of the y axis in each plot is different, in order to better display the variability of the effect in different conditions. The light blue diamonds show the average, which corresponds to the datapoints plotted in panels A–C.

In the numerosity task (Fig. 3A), we observed robust effects of both duration and size, although in opposite directions. While duration had a congruent effect (i.e., the longer the duration, the higher the perceived numerosity), size had an opposite, repulsive effect: the bigger the size of the dots, the lower the perceived numerosity. The LME test (adjusted-R^2^= 0.68), indeed, showed a significant interaction between ratio and magnitude (b = 20.96, t = 5.81, p < 0.001). This interaction was followed up with simpler LME tests considering each interfering dimension separately. The results of these additional tests showed that both duration (adj-R^2^= 0.64, b = 5.47, t = 2.64, p = 0.009) and size (adj-R^2^= 0.71, b = -15.49, t = -4.69, p < 0.001) induced significant biases on numerosity judgments. In the duration task (Fig. 3B), we observed again a significant interaction between ratio and magnitude (adj-R^2^= 0.53, b = -12.02, t = -2.57, p = 0.011), this time suggesting that size had a stronger influence on duration compared to the effect of numerosity on duration. Two follow-up LME tests, however, showed significant congruent biases induced by both numerosity (adj-R^2^= 0.44, b = 7.28, t = 2.42, p = 0.017) and size (adj-R^2^= 0.51, b = 19.29, t = 4.57, p < 0.001). Finally, looking atFigure 3C, it is clear that size was the magnitude most resistant to biases from other dimensions. Although weaker, the LME test (adj-R^2^= 0.66) showed a significant main effect of ratio (b = 4.45, t = 3.08, p = 0.002), no main effect of magnitude (b = -1.07, t = -0.32, p = 0.75), and no interaction (b = 0.12, t = 0.07, p = 0.94).Figure 3E–Jshows the individual measures of the magnitude integration effect across the group, with each panel corresponding to each effect in each task. As shown by the figure, the strength of the effect varies between different participants, but its direction (i.e., overestimation for increasing magnitude, except for the effect of size on numerosity) is consistent across the majority of participants (the directionality is not shown in the figure for the ease of visualization). For instance, the effect of numerosity on either duration or size worked consistently on 75–85% of the participants (respectively). The effect of duration had a consistent (positive) direction in 80% and 75% of participants, respectively in the numerosity and size task. Finally, the effect of size on numerosity and duration worked consistently (either in the negative or positive direction) in 90% and 75% of the participants, respectively. Overall, the behavioral results of the classification task showed robust and systematic mutual biases across all the dimensions tested, albeit with some partial asymmetries.

To assess the participants’ precision in the task, we considered the Weber fraction (WF; computed as the ratio of the just noticeable difference and the PSE), which is shown inFigure 3D. On average, size showed the lowest WF (0.18 ± 0.08), suggesting the highest precision in the task, followed by numerosity (0.24 ± 0.10), and finally duration (0.52 ± 0.32), which was the most difficult dimension to judge. A one-way repeated-measure ANOVA (with factor “task”) confirmed that the WFs across the three types of task are significantly different (F(2,38) = 18.59, p < 0.001), in line with previous studies (Fornaciai et al., 2023).

**Fig. 4.**
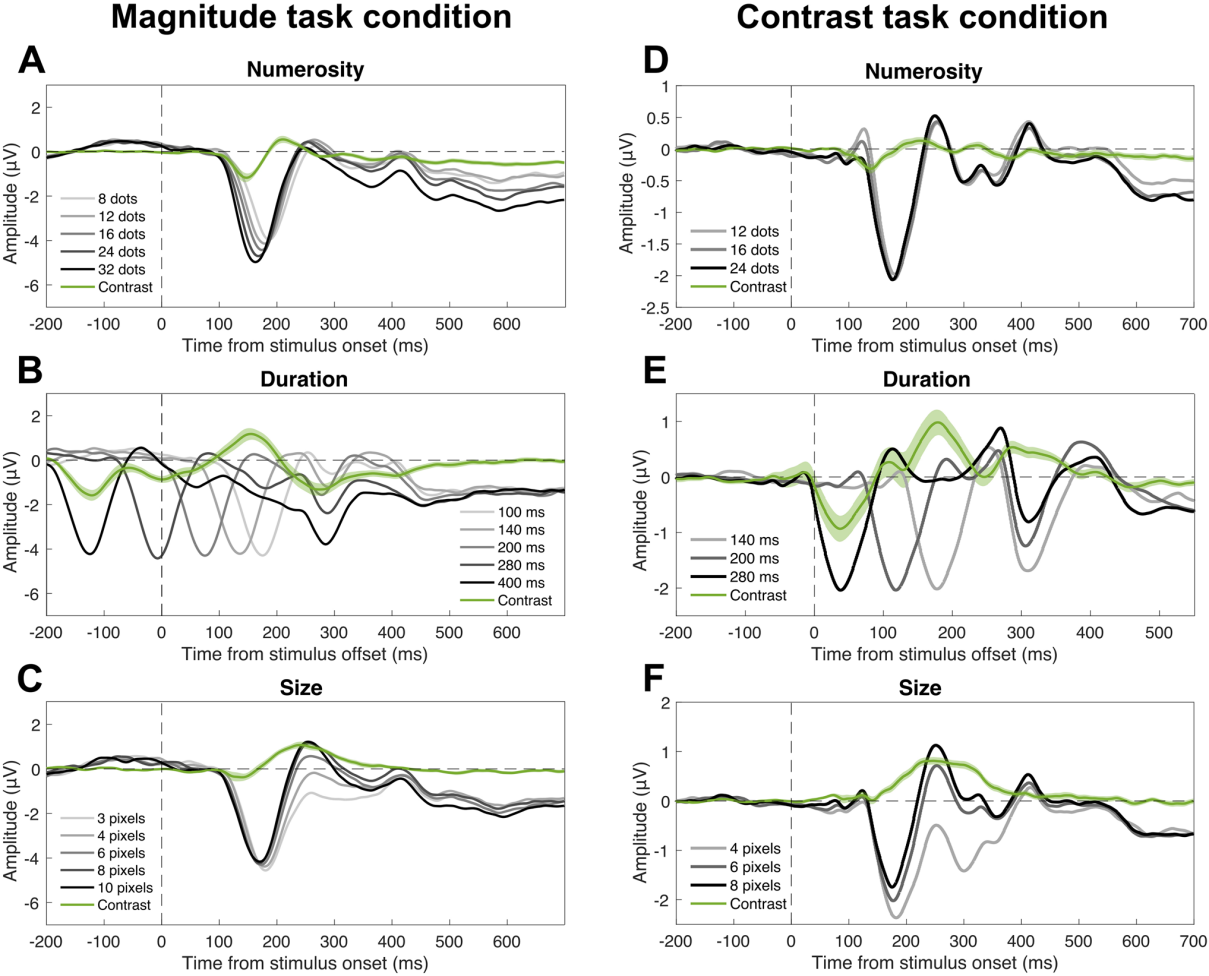
Event-related potentials (ERPs) evoked by each magnitude in the two experimental conditions. (A–C) Data from the magnitude task condition. (D–F) Data from the contrast task condition. (A) ERPs evoked by the stimulus numerosity in the magnitude task condition. (B) ERPs evoked by the stimulus duration in the magnitude task condition. (C) ERPs evoked by item size in the magnitude task condition. (D) ERPs evoked by the stimulus numerosity in the contrast task condition. (E) ERPs evoked by the stimulus duration in the contrast task condition. (F) ERPs evoked by item size in the contrast task condition. In all panels, the green wave indicates the linear contrast of the ERPs. Note that while numerosity and size ERPs were time-locked to the onset of the stimuli, ERPs corresponding to duration were re-aligned to the offset. The zero in (B) and (E) thus indicates the offset of the stimuli. The vertical dashed line indicates the onset or offset of the stimuli. The horizontal dashed line indicates the zero of the amplitude scale. All the ERPs are the average of signals from channels Oz, O1, and O2. The shaded area around the green contrast wave represents the SEM.

After assessing the behavioral effects, we went on and addressed the neural signature of magnitude integration. First, we plotted the event-related potentials (ERPs) evoked by the different levels of each magnitude, irrespective of the task performed. This was done to get an initial idea of how the different magnitude dimensions modulated the brain responses to the stimuli. This is particularly important in the case of duration, since the alignment of ERPs to the offset of the stimuli might introduce spurious results due to the mismatch of the onset response, which needs to be considered in order to contextualize the significance of effects at different latencies. The ERPs are shown inFigure 4A–C. In the case of numerosity (Fig. 4A), we observed a first negative peak of numerosity-sensitive responses at 150–200 ms after stimulus onset, followed by weaker but more sustained responses throughout the epoch. In the case of duration, aligning the waves to the offset of the stimuli, indeed, created a misalignment of the onset responses, which introduced spurious effects that are clearly visible inFigure 4B. In other words, a seemingly large deflection in the contrast amplitude is actually driven by a peak in amplitude of a single level of duration, rather than a consistent peak present at all levels of duration. However, we also observed a large modulation at around 300 ms after stimulus offset that seems to genuinely reflect duration, as it involves a deflection evident at all the levels of duration. Finally, brain responses sensitive to item size (Fig. 4C) showed a main peak at around 250 ms, with a large positive deflection. Although the effect of size starts at the first negative deflection at around ~180 ms, where bigger size is seemingly associated with smaller amplitudes, the effect at following latencies shows higher positive amplitudes for bigger sizes. The large positive peak of the contrast amplitude (green wave inFig. 4C), encompassing both the negative and positive peak, suggests that the two effects may not be independent, but possibly reflect similar more positive (or less negative) amplitudes for increasing size.

**Fig. 5.**
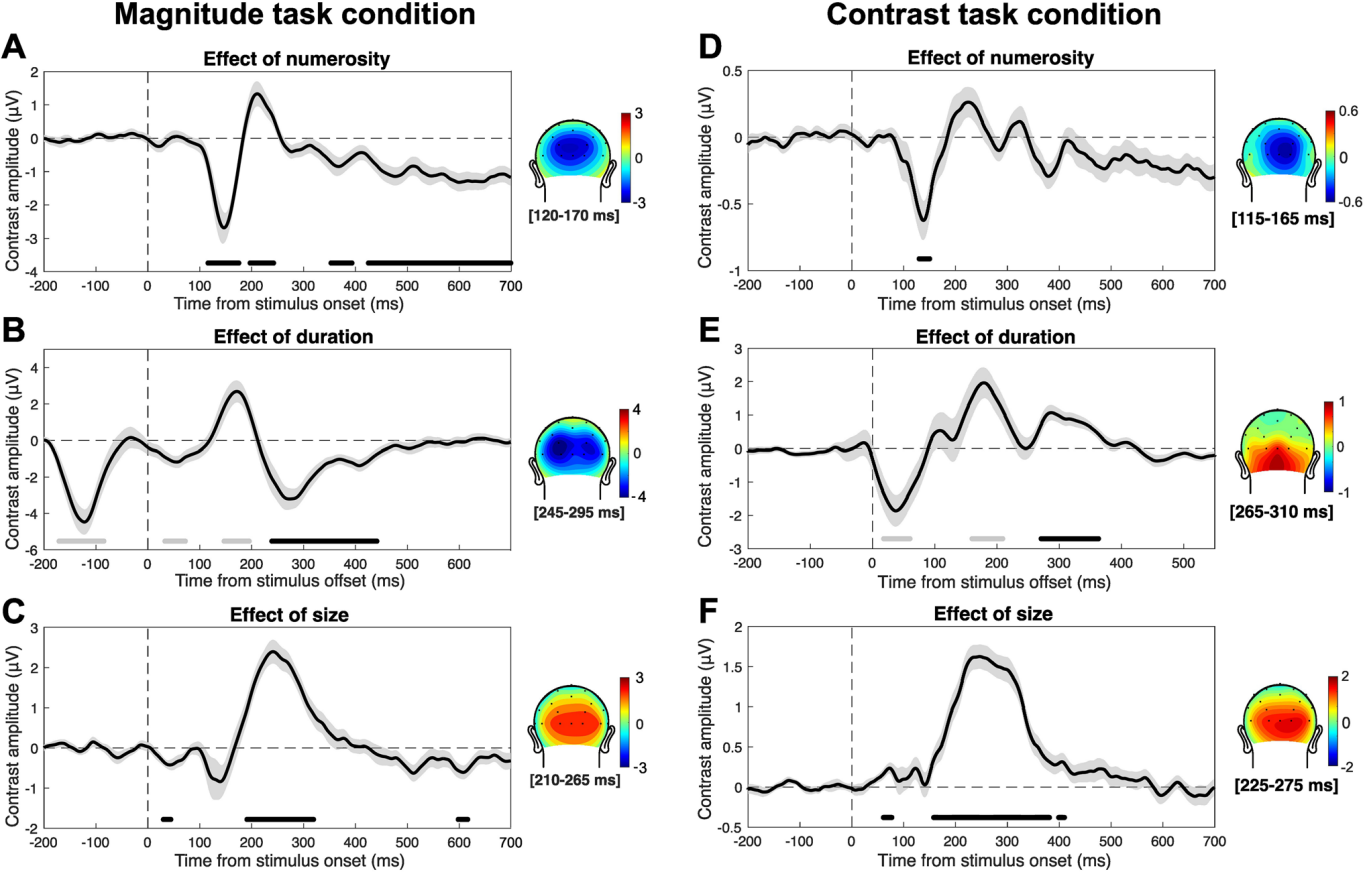
Average contrast amplitude reflecting the neural effects of the three magnitudes. (A–C) Data from the magnitude task condition. (D–F) Data from the contrast task condition. The contrast amplitude in this case was computed as the difference between the extreme levels of each “interfering” magnitude, separately for each level of each magnitude. For instance, the effect of numerosity on duration was computed as the difference between ERPs corresponding to 100 ms/32 dots and 100 ms/8 dots, and so on for the other levels of duration. The same was done for the effect of numerosity on size, and the resulting contrasts were averaged together to increase the signal-to-noise ratio. A similar procedure was then used to compute the effect of duration and the effect of size. (A) Contrast amplitude reflecting the effect of numerosity in the magnitude task condition. (B) Contrast amplitude reflecting the effect of duration in the magnitude task condition. (C) Contrast amplitude reflecting the effect of size in the magnitude task condition. (D) Contrast amplitude reflecting the effect of numerosity in the contrast task condition. (E) Contrast amplitude reflecting the effect of duration in the contrast task condition. (F) Contrast amplitude reflecting the effect of size in the contrast task condition. The black lines at the bottom of the plots mark the significant latency windows assessed with a series of FDR-corrected one-sample t-tests. In panel B and E, the significant latency windows driven by the onset response (i.e., spurious results due to the misalignment of the onset) have been marked in grey and not considered for further data analysis. The vertical dashed line indicates the onset or offset of the stimuli. The horizontal dashed line indicates the zero of the amplitude scale. The shaded area around the wave represents the SEM. The topographic plots besides each panel show the distribution of scalp activity in a 50-ms window around the main peak of each wave. All waves shown in the figure reflect the average of signals from channels Oz, O1, and O2.

To better address the significance of magnitude-sensitive brain responses, we computed a measure of ERP contrast based on the difference between the extreme levels of the interfering dimensions’ ranges. Such a measure of contrast (or difference) across ERPs, indeed, provides a more direct index of the sensitivity of brain responses to the different magnitudes, and makes it easier to assess the extent to which the amplitude of brain responses reflects the modulation of magnitude information. Specifically, for each level of each magnitude, we contrasted the ERPs as a function of the extreme levels of the interfering dimensions. For example, to compute the effect of numerosity, we subtracted the ERP corresponding to the combination of 100 ms and the two extreme levels of numerosity (8 and 32 dots). The same subtraction was performed for the combination of 140, 200, 280, and 400 ms and the extreme levels of numerosity. The same was done for the combination of each level of size and the extreme levels of numerosity. The effects of duration and size were computed in the same way by switching the dimension. The average of this contrast measure, reflecting the effect of the different magnitudes in driving ERPs, is shown inFigure 5. Additionally,Figure 5shows the topographic distribution of the contrast amplitude in a 50-ms window around the main peak of each corresponding contrast wave. To assess the significance of the contrast amplitude, we performed a series of one-sample t-tests against zero, corrected for multiple comparisons with false discovery rate (FDR; q = 0.05). When reporting the results below, we indicate the range of t-values and FDR-adjusted p-values as [min, max].

The numerosity-sensitive brain responses (Fig. 5A) showed four significant latency windows. The strongest effect was observed at a negative deflection at 120–175 ms (t = [-6.97, -2.41], p = [<0.001, 0.049]) after stimulus onset, which was around the peak of contrast amplitude (-2.7 μV) observed at 145 ms after stimulus onset. This peak was followed by additional significant windows at 200–240 ms (t = [2.45, 3.62], p = [<0.001, 0.046]), 355–390 ms (t = [-3.18, -2.41], p = [0.012, 0.048]), and 425–700 ms (t = [-5.61, -2.47], p = [0.001, 0.044]) after stimulus onset. Regarding the effect of duration on ERPs (Fig. 5B), we observed four significant latency windows. The first one was observed before stimulus offset, spanning from -170 to -85 ms (t = [-6.55, -2.58], p = [<0.001, 0.049]). Then, we observed two relatively early windows at 30–70 ms (t = [-3.66, -2.58], p = [0.007, 0.049]) and 145–195 ms (t = [2.58, 4.50], p = [0.003, 0.049]). Looking at the ERPs shown inFigure 4B, these three latency windows, however, appear to be driven each by a single duration level, due to the onset responses (i.e., only one wave shows a deflection while the others are flat). Such responses cannot thus be considered as genuine correlates of duration, but are spurious effects due to the re-alignment of brain waves to the offset of the stimuli. A more genuine peak of activity driven by duration was instead present at 270 ms after the offset (-3.2 μV), and we observed a significant latency window around this peak, spanning 240–440 ms (t = [-5.99, -2.60], p = [<0.001, 0.048]). An alternative possibility, however, is that such a peak at ~300 ms might be driven by differences occurring earlier in the ERPs (for instance at the onset response), propagating to later latencies and amplified during the interval between the onset and the offset of the stimulus. To assess this possibility, we time-locked the ERPs corresponding to the different durations to the onset of the stimuli, and assessed potential differences at the onset response (data not shown). The onset response peaked at 175 ms, and no difference was observed in the amplitude at such a peak (LME regression, adj-R^2^= 0.98, b < 0.001, t = -0.06, p = 0.95). This shows that the peak at 300 ms post-offset does not reflect differences in the level of amplitude at the onset response. This peak, thus, more likely reflects a genuine correlate of duration processing, like an accumulation of information over time (e.g., see alsoErnst et al., 2017). Finally, in the case of size (Fig. 5C), the peak of activity was observed at 240 ms (2.4 μV). The largest significant latency window was observed around this peak, spanning 190–320 ms (t = [3.14, 8.44], p = [<0.001, 0.049]). Two additional, smaller significant windows were observed at very early latencies (30–45 ms; t = [-4.04, -3.23], p = [0.009, 0.042]), and at later latencies (600–615 ms; t = [-3.59, -3.16], p = [0.022, 0.048]). In all cases, the topographic distribution of scalp activity around the peaks (plotted besides each panel,Fig. 5A–C) showed a posterior distribution consistent with activity in the occipital cortex.

**Fig. 6.**
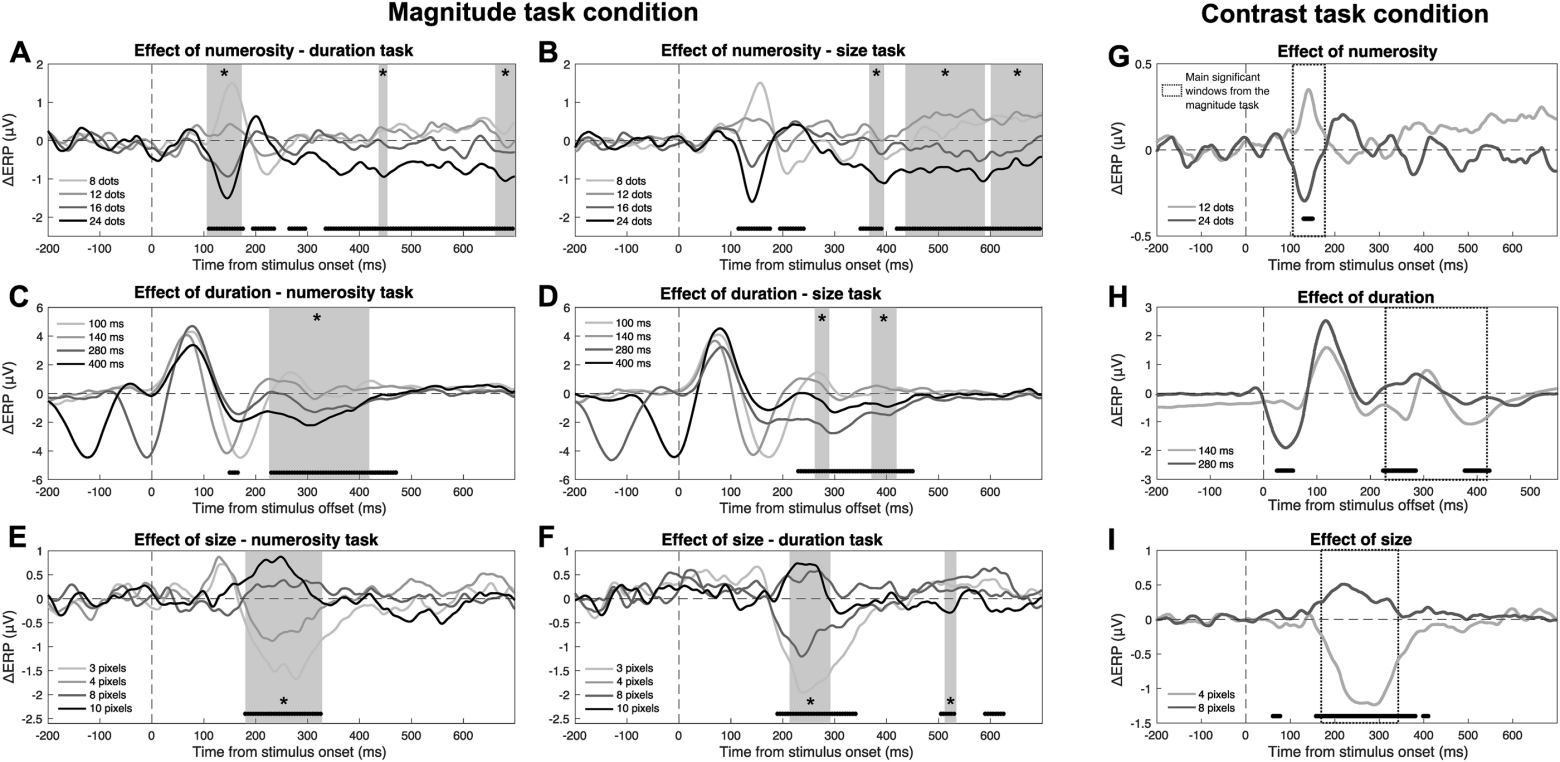
ΔERP measures and relationship with the behavioral effect. (A–F) Data from the magnitude task condition. (G–I) Data from the contrast task condition. (A) ΔERP measures reflecting the effect of numerosity in the duration task. (B) Effect of numerosity in the size task. (C) Effect of duration in the numerosity task. (D) Effect of duration in the size task. (E) Effect of size in the numerosity task. (F) Effect of size in the duration task. (G) ΔERP measures reflecting the effect of numerosity in the contrast task condition. (H) Effect of duration in the contrast task condition. (I) Effect of size in the contrast task condition. The black lines at the bottom of the plots mark the latency window where we observed a significant difference in ΔERP as a function of the different levels of the magnitude. The vertical dashed line indicates the onset or offset of the stimuli. The horizontal dashed line indicates the zero of the amplitude scale. The grey shaded areas marked with stars indicate the latency windows where we observed a significant relationship between ΔERP and the behavioral effect (ΔPSE). The dotted boxes in (G–I) (contrast task condition) show the main latency windows whereby the brain responses in the magnitude task condition predicted the behavioral effect. All waves shown in the figure reflect the average of signals from channels Oz, O1, and O2.

Our main goal in the magnitude task condition was, however, to identify the latency windows whereby the modulation of brain activity predicts the magnitude integration bias observed behaviorally. We then further computed two measures of the effect that could be related to each other in data analysis: ΔPSE, reflecting the behavioral effect, and ΔERP, reflecting the neural effect of magnitudes. This is very important, since individual ERPs corresponding to different levels of magnitudes are very difficult to relate to the behavioral effect, which is instead computed as a difference in perceived magnitude across different levels of the stimuli. In order to assess the relationship between the neural and behavioral effect of magnitude integration, we thus needed equivalent measures that can be related to each other and tested for statistical significance. These measures were computed by subtracting either the PSE or the ERP amplitude of each level of the interfering magnitudes from the PSE/ERP corresponding to the middle, reference level. For the ΔERP, this measure was computed at each time point throughout the epochs, and separately for the different types of task (seeFig. 6). Doing so, we thus computed the effect of numerosity in the duration and size task (Fig. 6A, B), the effect of duration in the numerosity and size task (Fig. 6C, D), and the effect of size in the numerosity and duration task (Fig. 6E, F).

To address the relationship between neural and behavioral measures of magnitude effects, we first looked for latency windows showing a significant modulation of ΔERP as a function of the different levels of the magnitudes. To do so, we performed a series of LME tests individually for the effect of each magnitude in each task. In the LME model, we entered the ΔERP as the dependent variable, the ratio of each magnitude level with the middle level as the predictor, and the subjects, as well as the ratio, as random effects. The LME tests were performed across a series of 10-ms windows with a 5-ms step, in a sliding-window average fashion. To control for multiple comparisons, we again applied an FDR procedure with q = 0.05. Clusters of less than three consecutive significant tests (after FDR) were not considered. The results of these tests are shown with black lines at the bottom of each plot inFigure 6, marking the significant latency windows.

After identifying the latencies showing a significant modulation of ΔERP, we looked for a relationship between ΔERP and ΔPSE within these windows. We thus performed a series of LME tests (10-ms windows with 5-ms step), including ΔPSE as the dependent variable and ΔERP as the predictor. The subjects and the ΔERP were also added as random effects. The effect of numerosity on duration (Fig. 6A) showed three windows whereby the modulation of ΔERP could predict the behavioral effect (marked with grey shaded areas in the figure), a larger early window at 110–170 ms, followed by two smaller windows at 440–450 ms and 665–695 ms (b = [0.007, 0.010], t = [2.04, 3.68], p = [<0.001, 0.044], adj-R^2^= [0.77, 0.79]). The effect of numerosity on size (Fig. 6B) showed again three significant windows, but clustered at later latencies: 370–390, 445–585, and 605–695 ms (b = [0.087, 0.180], t = [2.05, 4.12], p = [<0.001, 0.024], adj-R^2^= [0.78, 0.83]). The effect of duration on numerosity (Fig. 6C) showed a single large significant window, spanning 230–415 ms (b = [0.301, 0.597], t = [2.30, 3.90], p = [<0.001, 0.031], adj-R^2^= [0.79, 0.83]). The effect of duration on size (Fig. 6D) showed two smaller windows, with a timing generally consistent with the effect on numerosity: 265–285 and 375–415 ms (b = [0.067, 0.088], t = [2.02, 2.79], p = [0.007, 0.046], adj-R^2^= [0.81, 0.82]). Finally, the effect of size on numerosity (Fig. 6E) showed a single, large significant window at 190–310 ms (b = [0.845, 1.078], t = [3.18, 6.48], p = [<0.001, 0.002], adj-R^2^= [0.92, 0.99]), while the effect of size on duration (Fig. 6F) showed two significant windows at 215–290 and 515–530 ms (b = [-0.009, 0.013], t = [-3.06, 2.12], p = [0.003, 0.041], adj-R^2^= [0.76, 0.79]). These results show that the behavioral effect of magnitude integration could be reliably predicted by the modulation of magnitude-sensitive responses in the different task types, providing a neural signature of the effect.

### 3.2 Contrast task condition

In the contrast task condition, participants watched a stream of dot-array stimuli modulated in numerosity, duration, and item size, and responded to occasional oddball stimuli defined by a lower contrast. No instruction suggested the participants to explicitly attend the magnitudes of the stimuli. This different protocol was thus designed to provide a cleaner index of the responses to the different magnitudes, not confounded by magnitude decision-making or other task-related processes. If magnitude integration arises from automatic perceptual processes, then we expected to observe a similar modulation of brain responses consistent with the timing observed in the magnitude task condition. Instead, if magnitude-related decision making is necessary for magnitude integration to occur, the modulation of brain responses linked to the behavioral effect should not occur during a different task, like the one focused on the contrast of occasional oddball stimuli that we used in this condition.

First, we assessed the ERPs corresponding to the different levels of the three magnitudes (Fig. 4D–F), similarly to what we did in the magnitude task condition. As in the case of the magnitude task, this is an important step especially when it comes to the responses to duration. Indeed, the misalignment of onset responses can introduce spurious effects that need to be evaluated to contextualize the significance of effects in terms of ERP amplitude. The overall pattern was largely consistent with that observed in the magnitude task (seeFig. 4A–C), with however some differences. Numerosity (Fig. 4D) showed an early positive deflection that we did not observe in the magnitude task condition, with the magnitude of the stimuli modulating the amplitude in the negative direction (i.e., the smaller the numerosity, the higher the positive deflection in response amplitude). Looking at the contrast amplitude (green wave inFig. 4D), such an opposite effect of numerosity might, however, be part of the subsequent modulation in the negative direction, showing larger negative amplitudes for increasing numerosity. Additionally, ERPs at later latencies showed a weaker modulation compared to the magnitude task. Duration (Fig. 4E) showed a similar deflection compared to the magnitude task, but the modulation of amplitude was in the opposite direction (i.e., see topographic plots besides the panels). Finally, size (Fig. 4F) showed instead ERPs consistent with the magnitude task. Again, the effect of size on ERPs starts with a negative deflection whereby bigger sizes seem to be associated with smaller amplitudes. However, also in this context, it is more likely that the earlier negative peak and the later positive peak form a continuum whereby increasing size is associated with more positive (or less negative) amplitudes—as suggested by the contrast amplitude (green wave inFig. 4F) showing a single deflection in the positive direction.

To better assess the significance of the magnitude-sensitive brain responses, we computed again a measure of contrast based on the difference between the extreme levels of each magnitude (Fig. 5D–F), as in the magnitude task condition. This was done in order to achieve a more direct index of sensitivity to magnitude, reflecting the extent to which the ERPs are modulated by the different levels of each magnitude dimension. The contrast amplitude was then tested with a series of one-sample t-tests against zero, corrected with FDR (q = 0.05). In the case of numerosity (Fig. 5D), we observed a significant early window (130–150 ms; t = [-4.34, -3.64], p = [0.026, 0.047]), showing a negative deflection consistent with the magnitude task (seeFig. 4A). The peak of activity in this window (-0.62 μV) was at 140 ms. Differently from the magnitude task, we did not observe significant latency windows later on in the epoch. The effect of duration (Fig. 5E) showed three significant windows: the first one at 20–60 ms (t = [-3.99, -2.68], p = [0.003, 0.049]), the second at 160–210 ms (t = [2.68, 4.34], p = [0.002, 0.049]), and the third at 270–360 ms (t = [2.71, 4.76], p = [0.002, 0.043]). Note, however, that similarly to the magnitude task condition, the first two significant windows appear to be mostly driven by the onset responses of individual durations, while the last window shows a consistent deflection in responses corresponding to all the different levels of duration (seeFig. 3E). The topographic plot showing the distribution of peak activity (Fig. 5E), thus, reflects this last latency window (peak at 290 ms, 1.05 μV). Differently from the magnitude task condition, however, the contrast amplitude here showed a positive, rather than negative, deflection. Similarly to the magnitude task condition, we also additionally assessed whether there may be earlier differences in the response to different durations time-locked to the onset, and especially at the onset response. The peak of the onset response was identified at 178 ms after stimulus onset, and again we did not observe any difference in the amplitude of ERPs corresponding to different durations (LME regression, adj-R^2^= 0.99, b < 0.001, t = -0.46, p = 0.64). Finally, size (Fig. 5F) showed a large main window at 155–380 ms (t = [2.62, 10.88], p = [<0.001, 0.048]), with a peak at 245 ms (1.62 μV) consistent with the effect of size in the magnitude task condition. In addition, we observed two additional, smaller windows at 60–75 and 400–410 ms (t = [2.62, 3.63], p = [0.005, 0.049]). Similarly to the magnitude task, the topography of peak amplitude over the scalp showed a posterior distribution consistent with the occipital cortex (see plots beside panel D–F).

In order to better compare the modulation of brain responses in the magnitude task and in the contrast task condition, we also computed the ΔERP measure (Fig. 6G–I). The relationship between the behavioral and neural measures of magnitude integration in the magnitude task was, indeed, evaluated using this measure. Here, we thus computed the same measure in order to assess whether the modulation of brain responses in the contrast task occurs at the same processing stages (i.e., latency windows) that showed a relationship with the behavioral effect. The ΔERPs were assessed with a series of paired t-tests, performed considering 10-ms windows (step = 5 ms) as in the magnitude task condition, corrected with FDR (q = 0.05). The overall timing of the significant windows was consistent with the contrast measure (seeFig. 5D–F). ΔERPs reflecting the effect of numerosity (Fig. 6G) showed a significant modulation at 130–150 ms (t = [3.64, 4.33], p = [0.025, 0.047]). The effect of duration (Fig. 6H) showed a modulation at 225–285 ms (t = [-6.91, -2.78], p = [<0.001, 0.049]), and at 380–420 ms (t = [-3.68, -2.79], p = [0.016, 0.049]) after stimulus offset. Finally, in the case of size (Fig. 6I), we observed the main modulation in a large window spanning 160–380 ms (t = [-10.87, -2.62], p = [<0.001, 0.049]). We again observed two smaller significant windows at 60–75 ms (t = [-3.63, -2.65], p = [0.005, 0.046]) and 400–410 ms (t = [-2.85, -2.62], p = [0.032, 0.049]). As a comparison,Figure 6G–Ishows with dotted boxes the latency windows where we observed a significant relationship between neural and behavioral measures of magnitude integration in the magnitude task condition (Fig. 6A–F). In all cases, we observed an overlap between the significant windows in the contrast and magnitude task condition.

### 3.3 Multivariate decoding analysis

To achieve a more direct comparison of magnitude-sensitive brain activity during the magnitude and contrast task, we performed a multivariate “decoding” analysis across the two experimental conditions. Indeed, while the evidence that we have gathered so far from the comparison of ERPs across conditions is mostly qualitative, the multivariate analysis can provide a quantitative measure of how similar the pattern of brain activity evoked by magnitudes in different conditions is. In the analysis, we trained a classifier (support vector machine) with data from the magnitude task condition, and tested its ability to decode magnitude-sensitive brain activity in the contrast task condition (Fig. 2). This training and testing direction was chosen to obtain a larger set of classification accuracy (CA) values, in order to achieve more robust and stable results when testing the statistical significance of the decoding. Indeed, the analysis was performed by training the classifier on a set of data points each formed by the average data of one participant. The classifier was then tested separately on the average data of each participant in the contrast task condition group, according to a leave-two-out procedure, that is, two datapoints each corresponding to a class entered into the analysis were left out from the training set, and two independent datapoints from the contrast task conditions were used for testing. This procedure thus resulted in a distribution of CA values corresponding to the number of participants in the contrast task condition. We did not run the analysis in the opposite direction (i.e., training with the contrast task data and testing with the magnitude task data) since, due to the different number of data points (i.e., due to the different number of participants in the two conditions), the results would be difficult to combine. SeeSection 2for more information about the decoding procedure. According to our hypothesis, if the brain responses related to magnitude processing and integration are similar irrespective of the task, then the classifier should be able to decode magnitude information from the contrast task data. Otherwise, if magnitude processing entails mechanisms specific to the task performed, no above-chance decoding should be observable. The ability of the classifier to decode magnitude information was evaluated based on the distribution of CA values, obtained across a series of small time windows (i.e., 15-ms window with 5-ms step) throughout the epochs. The distribution of CA values at each time window was then tested with a permutation (sign flipping) test to assess whether it was significantly higher than chance level (0.5; seeSection 2for more information). A depiction of the different steps of the analysis is shown inFigure 2, while the results are shown inFigure 7.

**Fig. 7.**
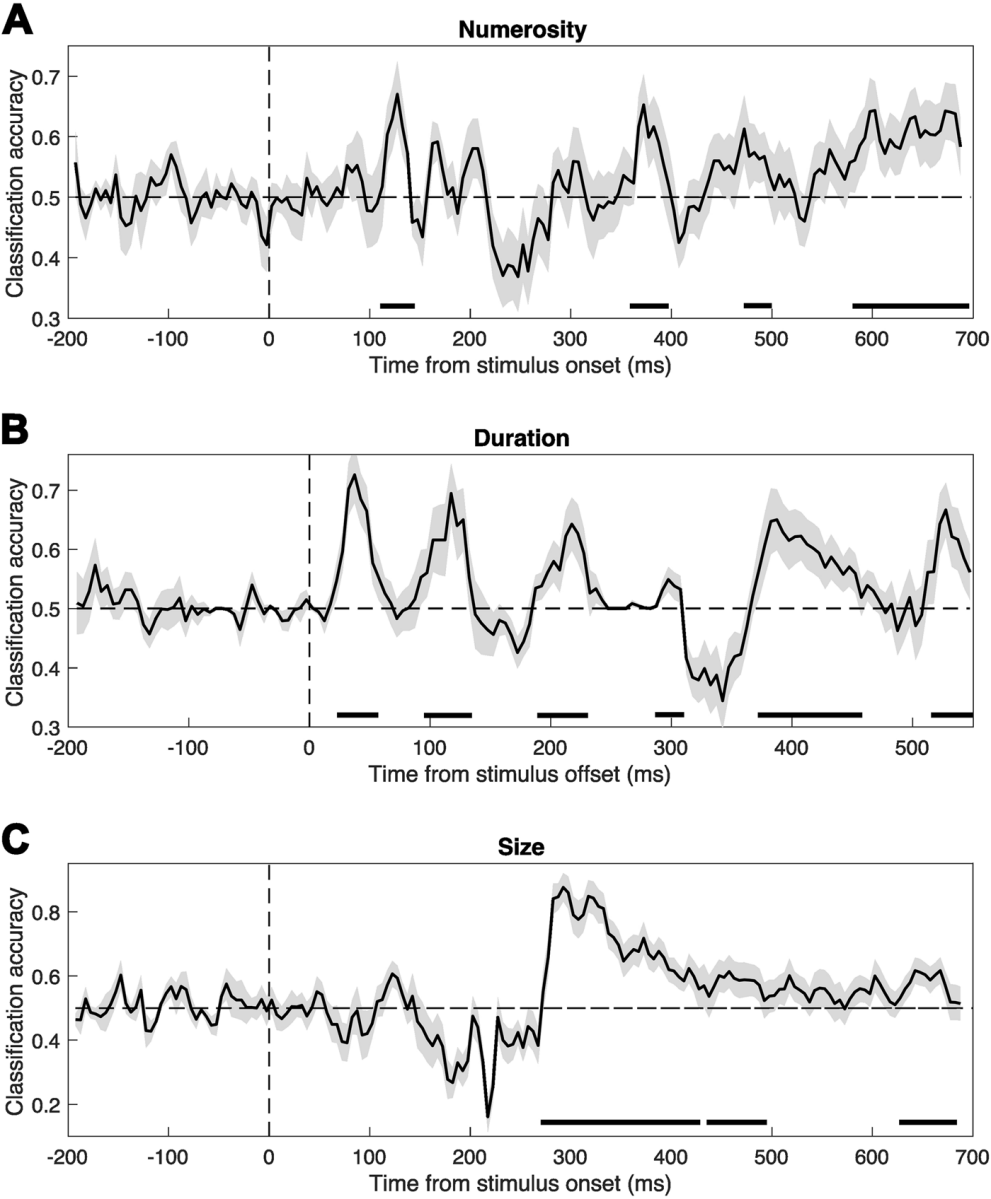
Results of the multivariate decoding analysis. The decoding analysis was performed by training a classifier on data from the magnitude task condition, and then tested on data from the contrast task condition, in order to achieve a more direct comparison of the magnitude-related brain processes in the two experimental conditions. (A) Classification accuracies obtained in the decoding of numerosity. (B) Classification accuracies obtained in the decoding of duration. (C) Classification accuracies obtained in the decoding of size. The horizontal dashed lines indicate the chance level (0.5). The vertical dashed lines mark either the time of stimulus onset for numerosity and size, or the time of stimulus offset for duration. The shaded area around the waves represents the SEM, which reflects the variability across the distribution of classification accuracy values obtained in the decoding procedure. The black lines at the bottom of the plot mark the latency windows where the decoding is significantly higher than chance level (0.5), as observed with a series of permutation tests.

Overall, the multivariate analysis revealed several latency windows in which magnitude-sensitive brain responses in the contrast task condition could be successfully predicted based on the training with the magnitude task condition data. Below, the results are reported in terms of the range of CAs observed (CA = [min, max]) and p-values of the permutation tests (p = [min, max]). In the case of numerosity (Fig. 7A), the analysis showed significant above-chance decoding at four latency windows. Namely, an early window spanning 110–145 ms after stimulus onset (CA = [0.57, 0.67], p = [<0.001, 0.010]), followed by later windows at 360–395 ms (CA = [0.59, 0.65], p = [<0.001, 0.049]), 475–500 ms (CA = [0.56, 0.57], p = [0.026, 0.049]), and 585–695 ms (CA = [0.57, 0.64], p = [<0.001, 0.036]). In the case of duration (Fig. 7B), we observed six significant latency windows, at 20–60 ms after stimulus offset (CA = [0.58, 0.73], p = [<0.001, 0.013]), 95–135 ms (CA = [0.62, 0.69], p = [<0.001, 0.017]), 185–235 ms (CA = [0.54, 0.64], p = [<0.001, 0.038]), 285–310 ms (CA = [0.53, 0.55], p = [0.004, 0.048]), 370–460 ms (CA = [0.56, 0.65], p = [<0.001, 0.039]), and 515–550 ms (CA = [0.58, 0.67], p = [<0.001, 0.035]). Finally, in the case of size (Fig. 7C), we observed three significant latency windows, at 270–430 ms (CA = [0.60, 0.85], p = [<0.001, 0.020]), 440–495 ms (CA = [0.58, 0.61], p = [0.008, 0.047]), and 630–680 ms (CA = [0.58, 0.62], p = [<0.001, 0.049]).

## 4 Discussion

In the present study we assessed and compared the signatures of magnitude integration in two different conditions: when participants are engaged in actively judging the magnitude of the stimuli, or when they passively watched a modulation of magnitude while attending another feature (i.e., contrast). The phenomenon of magnitude integration—that is, the mutual biases usually observed across different dimensions—is a hallmark of magnitude perception. Indeed, stimulus dimensions such as numerosity, duration, and size systematically interact with each other, leading to biases when judging them. Such mutual interactions have played a pivotal role in the development of influential theories like “a theory of magnitude” (ATOM;Walsh, 2003) and the “metaphor” theory (Casasanto & Boroditsky, 2008). However, the nature of this bias and its underlying mechanisms remain unclear.

Different mechanisms have been proposed to explain the interaction of magnitude dimensions. On the one hand, according to ATOM, the interaction would occur in perceptual processing due to the encoding of different dimensions with a common neural code (Walsh, 2003). In support of such a perceptual account of magnitude integration, we have recently shown that the effect relies on a mechanism similar to perceptual binding, inducing a positive bias across dimensions only when they are conveyed by the same stimulus (i.e., as opposed to magnitudes conveyed by separate, superimposed stimuli;Togoli, Bueti, et al., 2022). Recent neuroimaging studies, however, albeit showing common neural substrates, failed to provide evidence for a shared neural code (Borghesani et al., 2019;Hendrikx et al., 2024;Tsouli et al., 2022). According to the metaphor theory, on the other hand, the effect would instead arise at the conceptual or linguistic level, due to the use of “spatial” concepts to describe time (e.g., a “long” time;Bottini & Casasanto, 2013;Casasanto & Boroditsky, 2008; but seeWhitaker et al., 2022). This theory, however, relies on asymmetric effects across temporal and non-temporal dimensions, which depend on the type of stimuli used (Javadi & Aichelburg, 2012;Lambrechts et al., 2013;Togoli et al., 2021). Moreover, other authors proposed that magnitudes could interact during working memory maintenance, nudging each other while stored in memory (Cai et al., 2018;Cui et al., 2022), or as a bias during the response selection in comparisons tasks (Yates et al., 2012). Considering the results from these studies, whether magnitude integration across dimensions (e.g., numerosity, duration, and size) occurs at a perceptual or at a post-perceptual stage remains a debated topic.

In the present study, we addressed the nature of the magnitude integration effect by assessing a new prediction. Namely, a high-level effect hinging upon magnitudes concurrently held in memory (i.e., one magnitude biasing the memory of the other) or on active decision-making (i.e., one magnitude interfering with the response to another magnitude) should show a unique neural signature not present when the magnitudes are neither explicitly attended nor judged. Conversely, a perceptual effect is expected to occur in a more automatic fashion, independently from the relevance or judgment of magnitude. Thus, similar signatures should be observable with or without a magnitude judgment task.

Our behavioral results show systematic biases across the three magnitudes. First, numerosity is biased by both duration and item size. However, while duration shows a congruent effect (the longer the duration, the higher the perceived numerosity) as in previous studies (Javadi & Aichelburg, 2012;Togoli et al., 2021), size induces an opposite bias. Although different from the relationship between other dimensions, this result is consistent with previous studies showing that the effect of dot size on numerosity entails a negative effect, so that the larger the dot size, the lower the perceived numerosity (DeWind et al., 2015;Fornaciai et al., 2019). Duration is instead similarly affected by both numerosity and size in a congruent fashion, in line with previous studies (e.g.,Lambrechts et al., 2013;Xuan et al., 2007), although the latter exerts a stronger influence. Finally, size seems the dimension most resistant to integration biases, and shows only modest, albeit significant, influences from the other magnitudes. Size is also the dimension that is the easiest to judge (Fig. 3D), and the generally lower variability of responses might explain its robustness to biases. However, in a previous study from our group addressing trial-history effects in different magnitude dimensions (i.e., “serial dependence” effects;Fornaciai et al., 2023), size showed stronger biases compared to duration and numerosity, while again showing the highest precision. Thus, the perception of size does not seem intrinsically more resistant to biases, and the lower effect observed here might be a feature of magnitude integration effects rather than a general property. Considering the pattern of effects across dimensions, the results thus show some partial asymmetries, as some dimensions are more vulnerable to biases than others, in line with previous studies using similar stimuli (Togoli, Bueti, et al., 2022;Togoli et al., 2021).

In terms of event-related potentials, in the magnitude task condition we found robust brain responses to the different magnitudes. Overall, our analyses identified a set of latency windows that show the stronger peaks of activity driven by the different dimensions. Namely, around 150 and 250 ms after stimulus onset in the case of numerosity and size, and around 300 ms after stimulus offset for duration. Brain activity at these latency windows appears to be modulated by the different dimensions in a parametric fashion, according to the magnitude of the stimuli. Crucially, with just one exception (i.e., the effect of numerosity in the size task), brain activity at or around such peaks can significantly predict the bias observed behaviorally: the larger the brain responses, the stronger the magnitude integration bias. In the case of numerosity, the timing observed here (~150 ms) is consistent with numerosity-sensitive responses measured in previous studies. Although this timing is slightly earlier compared to the P2p component (~200 ms), that is, the ERP component most often associated with numerosity (Grasso et al., 2022;Libertus et al., 2007;Park et al., 2016;Temple & Posner, 1998), several studies also showed numerosity-sensitive responses at earlier latencies, starting at around 75–100 ms after stimulus onset (Fornaciai & Park, 2018;Fornaciai et al., 2017;Park et al., 2016). In the case of duration, previous studies highlight a variety of possible EEG correlates of duration processing, like the contingent negative variation (CNV;Damsma et al., 2021; but seeKononowicz & Penney, 2016), the N2 (Tonoyan et al., 2022), the P2 (Li et al., 2017), and the P3 (Cui et al., 2022;Ernst et al., 2017) ERP components. The timing shown in our results appears to be consistent with the results ofBenau et al. (2018), showing duration sensitivity at around 350 ms after stimulus offset. Finally, in terms of size, the timing of responses sensitive to the size of the items appears to be roughly consistent with previous results (Park et al., 2016) showing a peak at around 200 ms. Overall, these results, together with the results of previous studies, suggest that the processing of different dimensions unfolds with different dynamics, with peaks at different latencies. This supports the idea (Togoli et al., 2021) that each magnitude dimension affects other dimensions at different stages, depending on its specific processing dynamic. For instance, numerosity shows a relatively fast processing in the range of 75–200 ms after the onset of a stimulus (e.g.,Fornaciai et al., 2017;Park et al., 2016), so that it can potentially interfere with early processing stages relative to other dimensions like duration or size. Time perception, instead, requires the entire interval to unfold before a duration representation can be formed, so that its interference with other dimensions likely occurs at later processing stages after the offset of a stimulus.

Besides these differences across dimensions within each experiment, the crucial comparison concerns the responses to each magnitude across the different experimental conditions. In this context, the timing of magnitude-sensitive brain responses in the contrast task revealed similar evoked activity in most of the cases, closely mirroring the responses observed in the magnitude task condition. Especially in the case of numerosity and size, the peaks of magnitude-sensitive activity (i.e., ΔERP; compareFig. 6A–FwithFig. 6G–I) show, indeed, a one-to-one correspondence, with similar timing and polarity. In duration perception, however, although the timing and topography of responses is very similar, we observed ERPs with an opposite polarity. This may additionally suggest that while the processing of numerosity and size is largely invariant across the two conditions, the brain responses to duration may at least partially depend on the task relevance of this dimension. Namely, while the same duration processing stage seems to get engaged (i.e., as suggested by brain responses at the same latency and with the same scalp topography), actively attending the magnitudes of the stimuli may modulate how duration information is processed. This is not completely surprising, as duration shows different properties compared to the other dimensions (i.e., duration information needs to be accumulated, while the other dimensions can be processed from the onset), and the encoding of duration information is notoriously poorer in vision compared to other senses (e.g.,Alais & Burr, 2004;Cai & Connell, 2015).

Duration in this context seems, thus, to potentially engage different processes compared to the other dimensions. The timing of the duration effect, indeed, might be consistent with the engagement of post-perceptual processes, for instance involving an interference with the working memory traces of other magnitudes. Specifically, the timing of this effect is consistent with something similar to the P3 component, occurring after the offset of the interval. Such a component has been linked to the working memory representation of magnitude and the interference across different dimensions occurring in working memory (Cui et al., 2022). Together with the effect of attention and task-relevance discussed above (i.e., different ERP polarity in the two task conditions), our result may suggest the involvement of later processes beyond perception in driving the effect that duration exerts on other magnitudes. Such a later, higher-level interaction may be forced by the use of static stimuli creating a large mismatch between the processing of temporal and non-temporal dimensions (Togoli et al., 2021). Namely, due to the faster time course of numerosity and size processing (Fornaciai et al., 2017;Park et al., 2016), a duration representation formed after the offset of the interval may no longer be able to interfere with the perceptual processing of non-temporal dimensions, but only with later post-perceptual processes. An interesting goal for future studies is thus to address the neural signature of magnitude integration using dynamic stimuli in which dimensions such as numerosity and size unfold over time as well, making their processing time-course more similar to duration (seeLambrechts et al., 2013;Togoli et al., 2021,^2024^).

The lack of decision-making processes related to magnitude in the contrast task paradigm represents the major strength of this approach, as it allows to exclude the involvement of task-related brain processes concerning the maintenance and judgment of the different magnitudes (e.g., working memory maintenance and retrieval of magnitude information;Cai et al., 2018;Cui et al., 2022), and response biases (Yates et al., 2012). However, it also has the obvious limitation that magnitude integration could not be directly measured to confirm the effect. The striking similarity in the brain responses to the magnitudes, peaking at the same latencies where we demonstrated a relationship with the behavioral effect and showing the same scalp topography, nevertheless, provides evidence that magnitude integration likely occurs even in the absence of a magnitude task. While this comparison remains qualitative, the multivariate “cross-condition” decoding analysis provides quantitative evidence that the brain activity at several latency windows does not depend on the presence of a magnitude task. Indeed, the ability of the classifier to successfully decode the brain responses to magnitude across conditions shows that similar brain processes are engaged at specific time points, resulting in similar patterns of brain activity. In all cases, the latency windows showing above-chance decoding are largely consistent with the most important windows highlighted in the other analyses (e.g., in terms of ΔERP and its relationship with the behavioral effect). Namely, the timing of above-chance decoding in the case of numerosity (i.e., the 110–145 ms window), duration (i.e., 370–460 ms), and size (i.e., 270–430 ms) overlaps with similar windows showing a relationship between ERPs and behavior (in the magnitude task condition analysis), and with the effect of the different magnitudes in the contrast task condition. The analysis also highlights several other windows showing significant cross-condition decoding, suggesting that similar patterns of brain activity emerge at multiple processing stages across conditions, both early and late. The decoding analysis, thus, provides further evidence that magnitude processing entails patterns of brain activity largely independent from the task. In other words, with this analysis we demonstrate that the magnitude and contrast task condition not only entail similar magnitude-sensitive responses at the same processing stages, but also that such responses show very similar patterns of activity likely reflecting the same brain processes. In turn, this also suggests that the magnitude integration phenomenon—which is reflected by activity at such latency windows—likely takes place irrespective from the task, in an automatic and perceptually-driven fashion. Even in the case of duration, which shows ERP of different polarity in different tasks and a later timing potentially consistent with post-perceptual processes, the successful cross-decoding suggests at least partially similar processes occurring independently from decision making. Overall, according to this interpretation, the integration of different magnitudes and the relative bias would occur via perceptual processes affecting how we experience the different dimensions, and not only their memory traces or how they are judged—especially when it come to the effect of non-temporal dimensions. Namely, for instance, when we underestimate a duration because it is paired with a low numerosity we perceptually experience a shorter duration.

Another potential limitation of the present study concerns the use of partially different stimulation designs, as well as a slightly different preprocessing (i.e., larger epochs in the magnitude task in the initial preprocessing). In particular, in terms of stimulation procedure, the contrast task condition involved smaller magnitude ranges with fewer levels of magnitude, and a faster presentation rate. The two experimental conditions were, in fact, designed independently, in order to optimize each paradigm to the specific aim of the condition, rather than to have exactly the same stimuli and procedure. Such differences could, in turn, potentially affect the comparison between the different conditions, limiting our ability to find a general correlate of magnitude processing independent of the task. However, despite these differences, we observed very similar brain responses to the magnitudes in terms of ERPs, and patterns of brain activity generalizing across the two conditions and across independent groups of participants (in the multivariate analysis). It is important to note in this context that what we compared in our main analyses is not the absolute responses to individual levels of each magnitude—which, indeed, could have been difficult due to the different ranges—but measures of*sensitivity*to the different magnitude dimensions and their*effect*on brain responses. Namely, measures such as the contrast (Fig. 5) and the ΔERP (Fig. 6) reflect the difference in responses across different magnitude levels. The main property of such indexes that is expected to change depending on the range is their amplitude (e.g., 12 and 24 dots are expected to result in a smaller ΔERP compared to 8 and 32 dots; compareFig. 6A, BwithFig. 6G), while the timing and dynamics of brain responses should not be affected. We, however, never considered the absolute amplitude of responses in our analysis, as in this context it is not a particularly meaningful measure (i.e., due to the testing of independent groups of participants with likely different levels of mean ERP amplitude). Thus, although the use of different paradigms, and particularly the different stimulation ranges, represents an important limitation that needs to be taken into account, we believe that such differences do not confound our interpretation. The results themselves, in fact, demonstrate that very similar brain responses are evoked by the stimuli across the two tasks despite such differences. Nevertheless, using paradigms as similar as possible is probably a better strategy to compare different types of tasks, and future studies should minimize any difference to potentially achieve more robust results.

Differently from the present study, previous EEG results concerning magnitude integration (involving duration and length) suggested the involvement of working memory interference (Cui et al., 2022).Cui et al. (2022), indeed, observed effects of duration and length after the offset of the intervals, at ERP components usually associated with working memory maintenance, such as the P2 and P3b. While the effect of duration in Cui et al.’s work shows a timing consistent with the present results (~250–300 ms after stimulus offset), length has an effect at a much different timing (~300 ms after stimulus*offset*) compared to our earlier peak of responses to the size dimension (~250 ms after stimulus*onset*). Additionally, the scalp topography of the magnitude effects had a much more anterior distribution, peaking at parieto-frontal locations, as opposed to our results showing a posterior, occipital distribution. However, Cui et al. also employed much different stimuli: longer intervals (800–1,200 ms) and quite large lengths up to 15 degrees of visual angle. Considering the relatively long durations and the fact that the stimuli were marked only at the beginning (onset) and end (offset) point, it is not surprising that they engaged memory processes (e.g., see, for instance,Rammsayer & Lima, 1991). Both magnitudes in such a task, indeed, rely on the memory trace of the first marker presented rather than on sustained sensory stimulation, making it more likely that any interference would involve higher-level memory processes. Although the effect of duration on other magnitudes might involve working memory interference due to its much later timing, our results in terms of earlier perceptual effects (numerosity and size) do not conflict with such an interpretation. Perceptual and mnemonic interferences are, indeed, not mutually exclusive processes, and can both occur depending on the nature of the stimuli and paradigm used. Our results, however, show that magnitude interactions can be perceptual in nature when based on stimuli relying on sensory/perceptual processing rather than memory, at least for stimulus dimensions such as numerosity and size.

How would this perceptual interaction occur in the brain? Interestingly, our results show that the effects across the different magnitudes do not occur at a unique, generalized stage, but show different timings consistent with different brain processing stages. In other words, the interference between magnitudes seems to depend on the specific processing dynamics of each dimension (see alsoTogoli et al., 2021), rather than a common processing stage. Our results are thus not fully consistent with the idea of a generalized magnitude processing system, as proposed by the ATOM framework (e.g.,Walsh, 2003). Instead, the results seem more in line with recent findings of separate topographical cortical maps of different magnitudes, partially overlapping with each other (Fortunato et al., 2023;Harvey & Dumoulin, 2017;Harvey et al., 2013,^2015^;Hendrikx et al., 2024;Protopapa et al., 2019). Recently, indeed, it has been proposed that the interaction between different magnitudes could arise from the overlap of neural populations sensitive to different dimensions but without neural alignments across dimensions (Hendrikx et al., 2024;Tsouli et al., 2022), therefore arguing against the existence of a centralized mechanism or a common magnitude neural code (Walsh, 2003).

To conclude, our results show that the neural signatures of magnitude processing and integration are very similar whether participants explicitly attend and judge magnitude information or passively watch the modulation of different magnitude dimensions. This, in turn, suggests that similar brain processing stages are engaged irrespective of the task, and thus that magnitude integration occurs even in the absence of magnitude decision-making. One exception is possibly duration, that due to its nature and its much later processing might interact with other dimensions at post-perceptual stages, as an interference with working memory traces. Nevertheless, our results provide new evidence supporting the idea that magnitude integration can occur as a perceptual phenomenon independent from the task performed, affecting the phenomenological appearance of the stimuli, and not exclusively their memory traces or the way in which they are judged.

## Data Availability

All the data generated in the study described in this manuscript and the code of the experiments are available on Open Science Framework, at this link:https://osf.io/sn9h8/.
